# METTL3 Mediates Epithelial–Mesenchymal Transition by Modulating FOXO1 mRNA N^6^‐Methyladenosine‐Dependent YTHDF2 Binding: A Novel Mechanism of Radiation‐Induced Lung Injury

**DOI:** 10.1002/advs.202204784

**Published:** 2023-04-18

**Authors:** Yang Feng, Ping Yuan, Hongjuan Guo, Liming Gu, Zhao Yang, Jian Wang, Wei Zhu, Qi Zhang, Jianping Cao, Lili Wang, Yang Jiao

**Affiliations:** ^1^ State Key Laboratory of Radiation Medicine and Protection School of Radiation Medicine and Protection Medical College of Soochow University Suzhou 215123 China; ^2^ Department of Cardio‐Pulmonary Circulation Shanghai Pulmonary Hospital School of Medicine Tongji University Shanghai 200030 China; ^3^ Department of Respiratory Medicine Suzhou Science & Technology Town Hospital Suzhou 215153 China; ^4^ Department of Radiotherapy the Affiliated Jiangyin People's Hospital of Nantong University Jiangyin 214400 China; ^5^ Department of Radiotherapy the First Affiliated Hospital of Soochow University Suzhou 215006 China

**Keywords:** epithelial–mesenchymal transition (EMT), forkhead box O1 (FOXO1), ionizing radiation (IR), methyltransferase‐like 3 (METTL3), N^6^‐methyladenosine (m^6^A), radiation‐induced lung injury (RILI)

## Abstract

The biological roles of epithelial–mesenchymal transition (EMT) in the pathogenesis of radiation‐induced lung injury (RILI) have been widely demonstrated, but the mechanisms involved have been incompletely elucidated. N^6^‐methyladenosine (m^6^A) modification, the most abundant reversible methylation modification in eukaryotic mRNAs, plays vital roles in multiple biological processes. Whether and how m^6^A modification participates in ionizing radiation (IR)‐induced EMT and RILI remain unclear. Here, significantly increased m^6^A levels upon IR‐induced EMT are detected both in vivo and in vitro. Furthermore, upregulated methyltransferase‐like 3 (METTL3) expression and downregulated *α*‐ketoglutarate‐dependent dioxygenase AlkB homolog 5 (ALKBH5) expression are detected. In addition, blocking METTL3‐mediated m^6^A modification suppresses IR‐induced EMT both in vivo and in vitro. Mechanistically, forkhead box O1 (FOXO1) is identified as a key target of METTL3 by a methylated RNA immunoprecipitation (MeRIP) assay. FOXO1 expression is downregulated by METTL3‐mediated mRNA m^6^A modification in a YTH‐domain family 2 (YTHDF2)‐dependent manner, which subsequently activates the AKT and ERK signaling pathways. Overall, the present study shows that IR‐responsive METTL3 is involved in IR‐induced EMT, probably by activating the AKT and ERK signaling pathways via YTHDF2‐dependent FOXO1 m^6^A modification, which may be a novel mechanism involved in the occurrence and development of RILI.

## Graphical abstract

The ionizing radiation (IR)‐induced methyltransferase‐like 3 (METTL3) enhances the m^6^A level of forkhead box O1 (FOXO1) mRNA, which recruits YTH‐domain family 2 (YTHDF2) to downregulate FOXO1 expression. The downregulation of FOXO1 expression in turn activates the AKT and ERK pathways, ultimately facilitating the progression of IR‐induced epithelial–mesenchymal transition (EMT) during radiation‐induced lung injury (RILI).

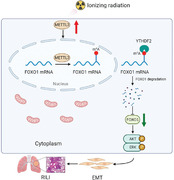

## 1 Introduction

Radiotherapy is the main therapeutic modality for thoracic malignancies such as lung, esophageal, and breast cancers either alone or in combination with other treatments.^[^ ^1^ ^]^ However, 5–15% of patients who undergo thoracic radiotherapy develop radiation‐induced lung injury (RILI), which is a critical dose‐limiting toxicity and a common complication.^[^ ^2^ ^]^ Alternatively, accidental or occupational ionizing radiation (IR) exposure, such as exposure caused by nuclear accidents and terrorist attacks, may also lead to RILI.^[^ ^3^ ^]^ RILI mainly manifests as acute radiation pneumonitis and pulmonary fibrosis,^[^ ^4^ ^]^ and can greatly reduce the quality of life and even lead to fatal respiratory insufficiency in patients. Unfortunately, the mechanisms of RILI remain incompletely understood, hampering the development of effective intervention strategies for RILI.^[^ ^5^ ^]^ Thus, it is necessary to elucidate the molecular mechanism of RILI to explore potential strategies for its prevention and treatment.

Epithelial–mesenchymal transition (EMT) is a vital pathological feature of chronic lung diseases.^[^ ^6^ ^]^ During EMT, epithelial cells progressively lose their epithelial features and gain mesenchymal fibroblast‐like characteristics.^[^ ^7^ ^]^ Originally discovered as a key mechanism of embryonic heart development, endothelial–mesenchymal transition (EndMT) is generally considered a subcategory of EMT because the endothelium is a special type of epithelium.^[^ ^8^ ^]^ Recently, numerous studies have reported that IR‐induced persistent inflammation prolongs lung epithelial and vascular endothelial cell damage, leading to EMT and/or EndMT and eventually pulmonary fibrosis.^[^ ^2^, ^9^ ^]^ Several studies suggest that transforming growth factor‐*β* (TGF‐*β*) activates EMT through the Smad2/3‐dependent pathway and the mitogen‐activated protein kinase (MAPK) pathway and then mediates RILI.^[^ ^10^ ^]^ Despite these findings, the precise mechanisms by which EMT and EndMT regulate RILI remain unclear.

N^6^‐methyladenosine (m^6^A) methylation is the most prevalent reversible modification in mammalian mRNAs^[^ ^11^ ^]^ and is introduced mainly by the m^6^A methyltransferase complex comprising methyltransferase‐like 3 (METTL3), methyltransferase‐like 14 (METTL14), and Wilms’ tumor 1‐associated protein (WTAP). The m^6^A modification is removed by *α*‐ketoglutarate‐dependent dioxygenase AlkB homolog 5 (ALKBH5) and fat mass and obesity‐associated protein (FTO).^[^ ^12^ ^]^ In addition, m^6^A modification is recognized by YT521‐B homology (YTH) domain‐containing proteins and heterogeneous nuclear ribonucleoprotein family members.^[^ ^12^ ^]^ Studies have confirmed that m^6^A modification is involved in various biological processes, such as the DNA damage response, heat shock response, T‐cell homeostasis, tumorigenesis, and metastatic and adipose tissue differentiation.^[^ ^13^ ^]^ The m^6^A modification has been reported to be associated with the progression of EMT in both normal^[^ ^14^ ^]^ and tumor cells.^[^ ^15^ ^]^ Intriguingly, recent reports have indicated that m^6^A methylation regulates the occurrence of pulmonary fibrosis.^[^ ^16^ ^]^ However, the biological function of m^6^A modification in the pathogenesis of RILI has not yet been reported, and whether m^6^A methylation is involved in regulating EMT during RILI remains unclear.

This study revealed a significant increase in METTL3‐mediated m^6^A modification in pulmonary tissues and cell lines undergoing IR‐induced EMT. METTL3 depletion inhibited EMT both in vitro and in vivo. Our results demonstrated that METTL3 enhances the m^6^A modification of forkhead box O1 (FOXO1) mRNA, thereby leading to YTH‐domain family 2 (YTHDF2) recruitment to support FOXO1 mRNA degradation and subsequently activating the AKT and ERK signaling pathways. Interventions targeting METTL3‐mediated m^6^A modification could effectively attenuate IR‐induced EMT and RILI, providing a potential prevention and therapeutic strategy for RILI by targeting the METTL3/FOXO1 axis.

## 2 Results

### 2.1 Elevated m^6^A RNA Levels Are Positively Correlated with IR‐Induced EMT In Vivo

**Figure 1:**
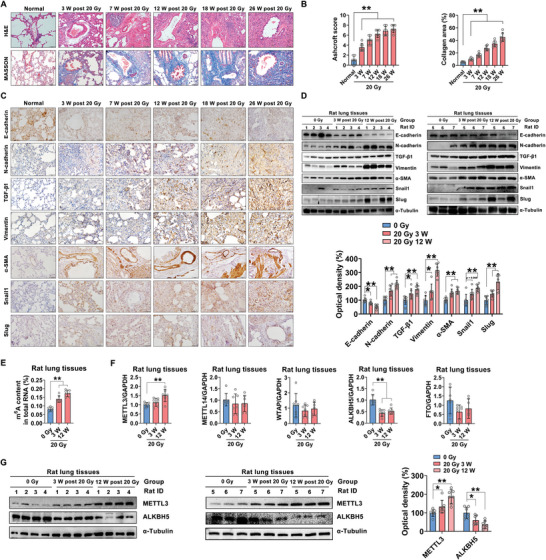
IR‐induced lung EMT in a rat model is regulated by m^6^A modification. A) Representative images of H&E and Masson's trichrome staining of rat lungs at 3, 7, 12, 18, and 26 weeks after irradiation (scale bar = 100 µm). B) Bar graph of the Ashcroft scores of H&E‐stained slides of rat lung tissues and quantification of the area of collagen deposition in rat lung tissues (*n* = 7 rats per group; mean ± SD; **p < 0.01; Student's t‐test). C) Immunohistochemistry was used to evaluate the E‐cadherin, N‐cadherin, TGF‐*β*1, Vimentin, *α*‐SMA, Snail1 and Slug levels in rat lung tissues (scale bar = 50 µm). D) Western blot and quantitative analyses of E‐cadherin, N‐cadherin, TGF‐*β*1, Vimentin, *α*‐SMA, Snail1 and Slug expression in rat lung tissues (*n* = 7 rats per group; mean ± SD; *p < 0.05, **p < 0.01; Student's t‐test). E) The total m^6^A level was elevated in rat lung tissues at 3 and 12 weeks post‐irradiation (*n* = 7 rats per group; mean ± SD; **p < 0.01; Student's t‐test). F,G) The mRNA and protein expression levels of m^6^A modification enzymes in rat lung tissues (*n* = 7 rats per group; mean ± SD; *p < 0.05, **p < 0.01; Student's t‐test).

To explore the pattern of m^6^A RNA modification during RILI, a rat lung fibrosis model was first established by unilateral pulmonary irradiation as previously described.^[^ ^17^ ^]^ As shown in **Figure** **1**A, after 20 Gy local irradiation, IR increased alveolar septal thickness and structural damage and aggravated the deposition of collagen, especially at 12, 18, and 26 weeks after IR exposure (Figure 1A,B). Moreover, an epithelial marker (E‐cadherin) was downregulated, and interstitial markers (N‐cadherin, TGF‐*β*1, Vimentin, *α*‐SMA, Snail1, and Slug) were upregulated in irradiated rat lung tissues compared with those in nonirradiated rat lung tissues, as observed by immunohistochemical staining (Figure 1C). These IR‐induced variations in the expression of EMT‐related markers were further confirmed by a Western blot analysis (Figure 1D). The above results indicated that IR‐induced EMT is closely associated with the occurrence and progression of RILI.

To identify the potential role of m^6^A RNA modification in IR‐induced EMT, the m^6^A levels of mRNAs in lung tissues from the RILI rat model were measured. The total m^6^A levels in irradiated lung tissues (3 weeks or 12 weeks after irradiation) were significantly higher than those in nonirradiated lung tissues, indicating that a correlation exists between m^6^A RNA modification and IR‐induced EMT during RILI (Figure 1E). Then, the expression of m^6^A methyltransferases (METTL3, METTL14, and WTAP) and m^6^A demethylases (ALKBH5 and FTO) in rat lung tissues during IR‐induced EMT was measured by qRT‐PCR. As shown in Figure 1F, elevated METTL3 expression was found in lung tissues at 12 weeks post‐irradiation compared to that in nonirradiated lung tissues. In contrast, the expression of ALKBH5 was reduced after IR exposure, while the expression of other m^6^A “writers” and “erasers” did not differ after IR exposure (Figure 1F). Furthermore, the time dependency of IR‐induced METTL3 expression and ALKBH5 suppression was identified, as shown in Figure 1G. The above results suggested that IR‐induced EMT is closely associated with the development of RILI, while increased m^6^A levels and expression levels of the m^6^A methyltransferase METTL3 and m^6^A demethylase ALKBH5 might be involved in IR‐induced EMT during RILI.

### 2.2 The m^6^A RNA Modification Is Involved in IR‐Induced EMT during RILI In Vitro

**Figure 2:**
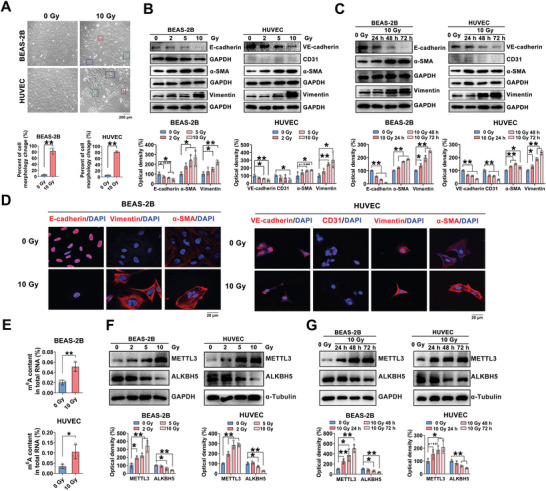
Variations in m^6^A RNA modification in IR‐induced EMT in vitro. BEAS‐2B cells and HUVECs were irradiated with a single dose of 0, 2, 5, or 10 Gy X‐rays. The cell morphology and levels of EMT‐associated protein markers were evaluated at 24, 48, and 72 h post‐irradiation. A) Representative images of cell morphology were acquired at 72 h after 0 or 10 Gy irradiation (scale bar = 200 µm). Cells with morphologic changes were counted in random microscopic fields, and the percentages of cells that became swollen (red outline), became elongated (green outline), or exhibited extended pseudopodia (blue outline) with respect to cells that retained a cuboidal morphology were calculated (*n* = 3; mean ± SD; *p < 0.05, **p < 0.01; Student's t‐test). B,C) Protein levels of E‐cadherin, VE‐cadherin, *α*‐SMA and Vimentin in pulmonary cells. (*n* = 3; mean ± SD; *p < 0.05, **p < 0.01; Student's t‐test). D) Immunofluorescence staining of E‐cadherin, VE‐cadherin, and *α*‐SMA (red) and DAPI staining (blue) in nonirradiated control and irradiated pulmonary cells at 72 h post‐irradiation (scale bar = 20 µm). E) The level of m^6^A‐modified RNA was elevated in pulmonary cells at 72 h after 10 Gy X‐irradiation (*n* = 3; mean ± SD; *p < 0.05, **p < 0.01; Student's t‐test). F) Western blot and quantitative analyses of the METTL3 and ALKBH5 protein levels at 72 h after different radiation doses (*n* = 3; mean ± SD; *p < 0.05, **p < 0.01; Student's t‐test). G) Protein expression of m^6^A modification enzymes in irradiated BESA‐2B cells and HUVECs at different time points (*n* = 3; mean ± SD; *p < 0.05, **p < 0.01; Student's t‐test).

Additionally, the variations in the m^6^A levels after irradiation were confirmed in vitro. Pulmonary cell lines, including BEAS‐2B cells, HUVECs and MLE‐12 cells, were exposed to 0, 2, 5, or 10 Gy X‐irradiation, and the cell morphology was observed at 0, 24, 48, and 72 h post‐irradiation. More than 80% of cuboidal pulmonary epithelial cells acquired a swollen and elongated morphology with extended pseudopodia, especially at 72 h after 10 Gy X‐irradiation (**Figure** **2**A; Figure S1A, Supporting Information). Correspondingly, significantly decreased E‐cadherin expression and increased Vimentin and *α*‐SMA expression were observed in the BEAS‐2B and MLE‐12 cells after IR exposure (Figure 2B,C; Figure S1B,C, Supporting Information). Consistent with this finding, the expression of endothelial markers (VE‐cadherin and CD31) and mesenchymal markers (Vimentin and *α*‐SMA) also varied in HUVECs (Figure 2B,C). In addition, the immunofluorescence staining confirmed the alterations in EMT‐associated markers (Figure 2D; Figure S1D, Supporting Information). Taken together, these data suggested that IR might induce EMT in lung epithelial cells and endothelial cells, especially at 72 h after 10 Gy X‐ray irradiation.

In addition, the total m^6^A levels were investigated in vitro in BEAS‐2B cells, HUVECs and MLE‐12 cells at 72 h after exposure to 10 Gy X‐irradiation. The m^6^A levels were significantly increased in the pulmonary cells that underwent EMT compared to those in nonirradiated control cells (Figure 2E; Figure S1E, Supporting Information). Our previous results showed that METTL3 or ALKBH5 was involved in EMT in a rat model of RILI (Figure 1). Then, the roles of METTL3 and ALKBH5 in EMT were identified in cell models of RILI. After exposure to a single dose of 0, 2, 5, or 10 Gy X‐rays, a dose‐dependent upregulation of METTL3 protein expression and suppression of ALKBH5 protein expression were observed in all pulmonary cell lines (Figure 2F; Figure S1F, Supporting Information). In addition, time‐dependent METTL3 upregulation and ALKBH5 downregulation were identified in BEAS‐2B cells, HUVECs and MLE‐12 cells after IR exposure (Figure 2G; Figure S1G, Supporting Information). Consistent with the in vivo results, these results showed that m^6^A RNA modification might be involved in EMT in vitro.

**Figure 3:**
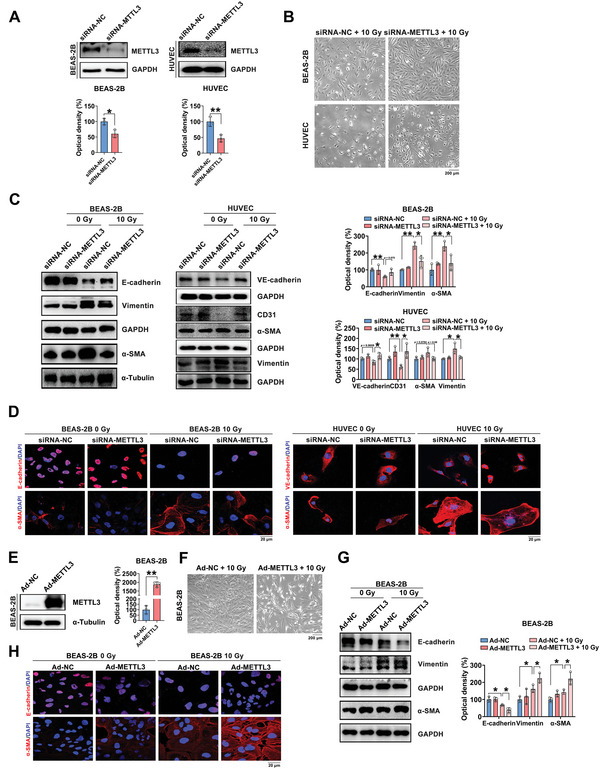
The m^6^A RNA modification mediates IR‐induced EMT during RILI in vitro. BEAS‐2B cells and HUVECs were transfected with control siRNA or METTL3‐specific siRNA with or without irradiation. A) Western blot and quantitative analyses of METTL3 expression in the two cell lines (*n* = 3; mean ± SD; *p < 0.05, **p < 0.01; Student's t‐test). B) Phase contrast micrographs of cells expressing METTL3‐specific siRNA and control siRNA at 72 h after 10 Gy irradiation (scale bar = 200 µm). C) Western blot and quantitative analyses of E‐cadherin, VE‐cadherin, CD31, Vimentin and *α*‐SMA expression in siRNA‐transfected cells at 72 h after 10 Gy irradiation (*n* = 3; mean ± SD; *p < 0.05, **p < 0.01; Student's t‐test). D) The expression of EMT‐related markers in siRNA‐transfected cells was detected using immunofluorescence at 72 h after irradiation (scale bar = 20 µm). BEAS‐2B cells were infected with Ad‐NC or Ad‐METTL3 prior to irradiation. E) Western blot and quantitative analyses of METTL3 expression in BEAS‐2B cells (*n* = 3; mean ± SD; **p < 0.01; Student's t‐test). F) Phase contrast micrographs of control and METTL3‐overexpressing BEAS‐2B cells at 72 h after 10 Gy irradiation (scale bar = 200 µm). G) Protein expression of EMT‐associated markers at 72 h after 10 Gy irradiation (*n* = 3; mean ± SD; *p < 0.05; Student's t‐test). H) Immunofluorescence staining was performed to detect the expression of E‐cadherin and *α*‐SMA in BEAS‐2B cells (scale bar = 20 µm).

To explore the function of m^6^A modification in regulating EMT, BEAS‐2B cells and HUVECs were used to establish METTL3 knockdown cell models by applying METTL3‐targeting siRNA (**Figure** **3**A). As shown in Figure 3B, the morphology of most METTL3 knockout cells was altered at 72 h after irradiation and ranged from a swollen, elongated morphology with extended pseudopodia to a cuboidal shape. Additionally, increased levels of epithelial/endothelial markers and decreased levels of interstitial markers were identified in the METTL3 knockdown BEAS‐2B cells and HUVECs after IR exposure (Figure 3C). Moreover, these morphologic variations were confirmed by immunofluorescence staining (Figure 3D). In contrast, these effects were abrogated, and EMT was even promoted by METTL3 overexpression in BEAS‐2B cells (Figure 3E–H). In addition, IR‐induced EMT was alleviated by the overexpression of the m^6^A demethylase ALKBH5 in MLE‐12 cells (Figure S1H–K, Supporting Information). Taken together, these results implied that m^6^A RNA modification mediates IR‐induced EMT in pulmonary epithelial cells.

### 2.3 METTL3 Regulates the Occurrence and Development of RILI by Triggering m^6^A Modification‐Mediated EMT In Vivo

**Figure 4:**
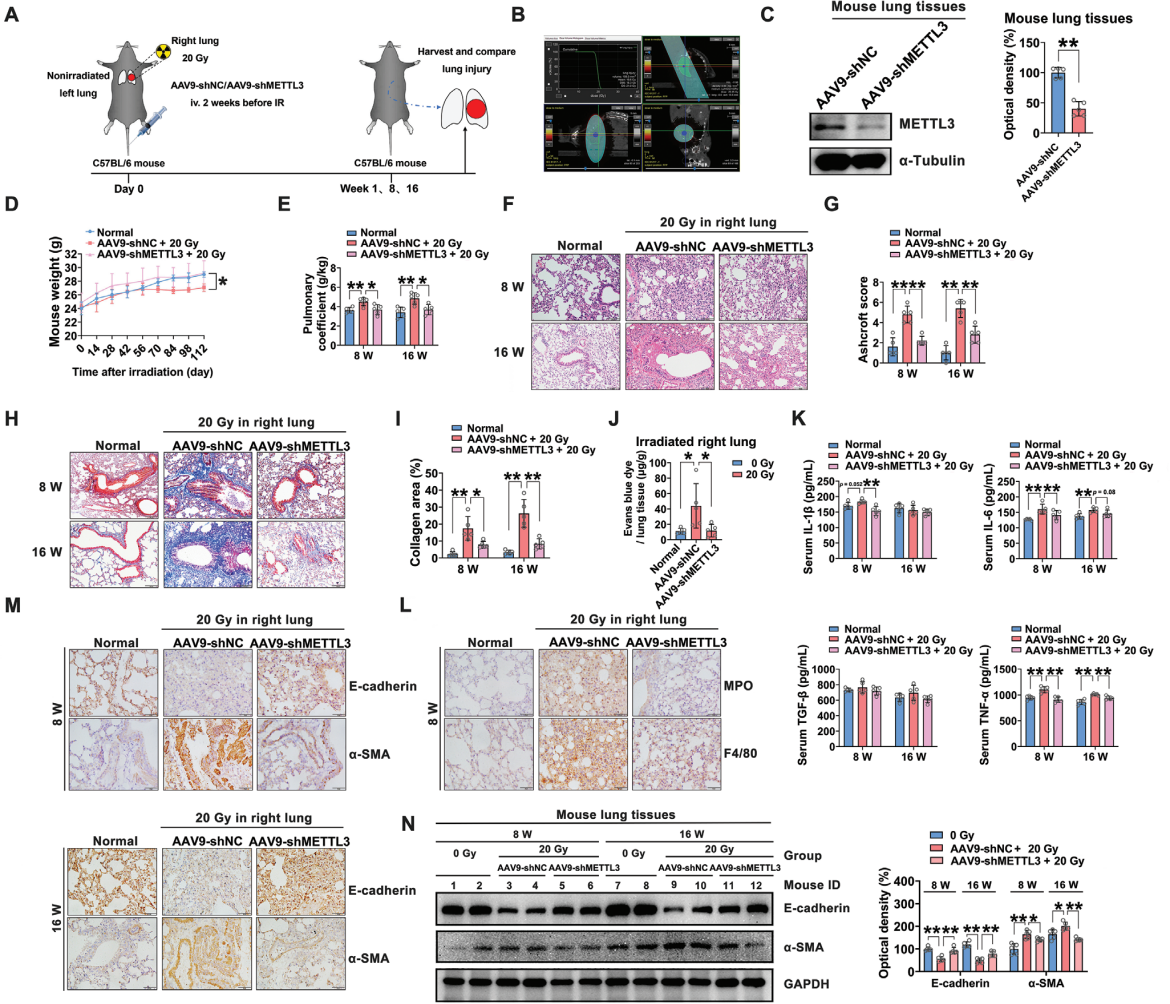
METTL3 regulates the occurrence and development of RILI by triggering m^6^A modification‐mediated EMT in vivo. A) Experimental scheme of the mouse treatment. The right lungs of mice remained unirradiated (control) or were irradiated with a single dose of 20 Gy X‐irradiation. Mice were injected with AAV9‐shNC or AAV9‐shMETTL3 via the tail vein two weeks before 20 Gy irradiation. Lung tissues were collected from the control and irradiated mice at 1, 8, and 16 weeks after irradiation (*n* = 5 mice per group). B) Target volume determination in mice. C) Western blot and quantitative analyses of METTL3 expression in lung tissues from the three groups of mice (*n* = 5 mice per group; mean ± SD; **p < 0.01; Student's t‐test). D) The weights of the mice in each group were monitored from week 0 to week 16 (*n* = 5 mice per group; mean ± SD; *p < 0.05; two‐way ANOVA). E) Line graph of the pulmonary coefficient in the different groups of mice following local irradiation. Pulmonary coefficient = lung weight (g)/mouse weight (kg) (*n* = 5 mice per group; mean ± SD; *p < 0.05, **p < 0.01; Student's t‐test). F) Representative H&E staining of right lung tissues from mice in different groups at 8 and 16 weeks after irradiation (scale bar = 100 µm). G) Bar graph of the Ashcroft scores of H&E‐stained slides of right lung tissues from mice (*n* = 5 mice per group; mean ± SD; **p < 0.01; Student's t‐test). H) Representative Masson's trichrome staining of the right lung from mice (scale bar = 100 µm). I) Quantification of the area of collagen deposition in right lung tissues from mice (*n* = 5 mice per group; mean ± SD; *p < 0.05, **p < 0.01; Student's t‐test). J) Evans blue staining was used to visualize pulmonary microvascular injury (*n* = 5 mice per group; mean ± SD; *p < 0.05; Student's t‐test). K) ELISA of the serum levels of IL‐6, IL‐1*β*, TGF‐*β*, and TNF‐*α* in mice from different groups in the RILI mouse model at 8 and 16 weeks post‐irradiation (*n* = 5 mice per group; mean ± SD; **p < 0.01; Student's t‐test). L) Right lung tissues were immunostained for F4/80 and MPO and counterstained with hematoxylin (scale bar = 50 µm). M) Immunohistochemical staining of E‐cadherin and *α*‐SMA in right lung tissues from the three groups of mice at 8 and 16 weeks post‐irradiation (scale bar = 50 µm). N) Lung protein levels of E‐cadherin and *α*‐SMA (*n* = 5 mice per group; mean ± SD; *p < 0.05, **p < 0.01; Student's t‐test).

To determine whether m^6^A modification can affect RILI in vivo, a model of RILI was established by unilateral pulmonary irradiation in mice with lung tissue‐specific METTL3 knockout accomplished by using intravenous injection of adeno‐associated virus 9 (AAV9)‐shMETTL3 (**Figure** **4**A–C). The pulmonary coefficient mainly reflects the degree of pulmonary edema.^[^ ^5^ ^]^ As shown in Figure 4D,E, a marked reduction in body weight and an increase in the pulmonary coefficient were observed in the AAV9‐shNC‐injected group. METTL3 depletion reduced the body weight loss and increased the pulmonary coefficient secondary to fibrosis (Figure 4D,E). After unilateral thoracic irradiation (20 Gy), thickening of the alveolar septum and infiltration of inflammatory cells were observed in the IR‐exposed lung tissues, in contrast to the sham‐irradiated counterparts (Figure 4F), which is consistent with the Ashcroft fibrosis score (Figure 4G). Histologically, the lung tissue‐specific METTL3 knockdown alleviated the formation of multifocal fibrotic lesions (Figure 4F,G). In the sham‐irradiated lung tissues, no significant histological difference was observed between the groups (Figure S2A,B, Supporting Information). As shown in Figure 4H,I, IR‐induced collagen deposition was obviously reduced by METTL3 knockdown in vivo. Comparatively, almost no significant difference was observed between the sham‐irradiated left lungs (Figure S2C,D, Supporting Information). In addition, IR‐induced vascular damage was evaluated by Evans blue staining,^[^ ^18^ ^]^ which revealed that the METTL3 deletion reduced the IR‐triggered vascular leakage observed at 1 week after IR exposure (Figure 4J). Moreover, almost no difference was observed between the sham‐irradiated left lungs (Figure S2E, Supporting Information).

Serum profibrotic cytokines are closely associated with the occurrence of pulmonary fibrosis.^[^ ^19^ ^]^ In the present study, the AAV9‐shMETTL3 treatment significantly decreased the serum levels of proinflammatory and profibrotic cytokines in irradiated C57BL/6 mice compared with those in vehicle‐treated control mice (Figure 4K). The infiltration of macrophages and neutrophils in lung tissues is an important pathological feature of RILI. F4/80 and MPO are biomarkers of macrophages and neutrophils, respectively.^[^ ^20^ ^]^ This work showed that the infiltration of macrophages and neutrophils in lung tissues was significantly increased at 8 weeks after IR exposure. Interestingly, the proportions of both F4/80‐ and MPO‐positive cells in the lung tissues with METTL3 knockdown were significantly lower than those in the lung tissues of mice injected with AAV9‐shNC (Figure 4L). The above data indicated that METTL3 knockdown may mitigate inflammation in irradiated lung tissues.

IR‐induced EMT has been considered closely associated with the development of pulmonary fibrosis.^[^ ^5^ ^]^ This work showed that E‐cadherin was progressively downregulated and that *α*‐SMA was significantly upregulated at 8 and 16 weeks after irradiation (Figure 4M). In the AAV9‐shMETTL3 treatment group, the pattern of *α*‐SMA upregulation and E‐cadherin downregulation was significantly reversed (Figure 4M). Additionally, using a Western blot analysis, METTL3 deletion was confirmed to inhibit EMT in irradiated lung tissues (Figure 4N). A similar protective role of excess ALKBH5 in ameliorating RILI was observed, while no such effect was observed in the unirradiated left lung tissues (Figure S3A–O, Supporting Information). These results demonstrated that a decrease in m^6^A modification can significantly inhibit the occurrence of early radiation‐induced pneumonia and late radiation‐induced pulmonary fibrosis.

### 2.4 FOXO1 Is a Potential Target of METTL3‐Mediated m^6^A Modification during RILI

**Figure 5:**
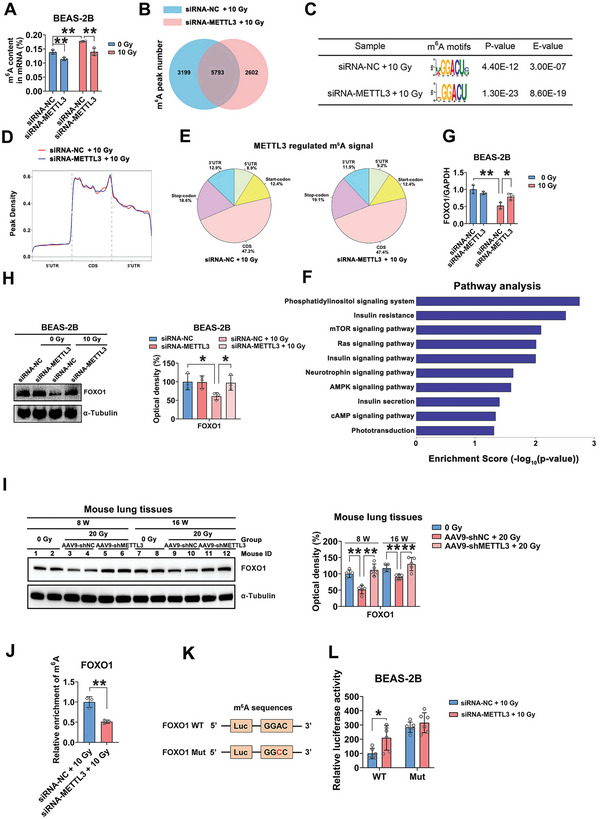
FOXO1 is a potential target of METTL3‐mediated m^6^A modification. A) m^6^A level of mRNA in BEAS‐2B cells with or without METTL3 knockdown (*n* = 3; mean ± SD; **p < 0.01; Student's t‐test). B) MeRIP‐seq identified 8992 and 8395 m^6^A peaks in the control and METTL3 knockdown BEAS‐2B cells, respectively. C) The GGAC motif was detected as the predominant consensus motif in both control and METTL3‐deficient cells. D) Density distribution of m^6^A peaks across mRNA transcripts in control and METTL3‐deficient cells. E) Proportions of m^6^A peaks distributed in the 5’UTR, start codon region, CDS, stop codon region, or 3’UTR across the entire set of mRNA transcripts. F) KEGG pathway enrichment analysis of hypomethylated m^6^A transcripts (*p* < 0.05 was used as the threshold for significant enrichment). G,H) Relative mRNA and protein expression of FOXO1 in BEAS‐2B cells with or without METTL3 depletion (*n* = 3; mean ± SD; *p < 0.05, **p < 0.01; Student's t‐test). I) FOXO1 protein expression in lung tissues in the RILI mouse model (*n* = 5 mice per group; mean ± SD; **p < 0.01; Student's t‐test). J) MeRIP‐qPCR analysis of the FOXO1 m^6^A level in irradiated BEAS‐2B cells with or without METTL3 knockdown (*n* = 3; mean ± SD; **p < 0.01; Student's *t‐test*). K) The WT or mutated m^6^A consensus sequence was fused to the firefly luciferase reporter. L) Luciferase vectors with WT or mutated FOXO1 were transfected into BEAS‐2B cells with or without METTL3 knockdown prior to irradiation. Relative luciferase activity was determined (*n* = 6; mean ± SD; *p < 0.05; Student's t‐test).

To delineate the molecular mechanism by which METTL3 regulates EMT during RILI, METTL3 knockdown or METTL3‐overexpressing BEAS‐2B cells with reduced (**Figure** **5**A; Figure S4A, Supporting Information) or elevated (Figure S4B, Supporting Information) m^6^A levels were generated. Then, methylated RNA immunoprecipitation (MeRIP) with an m^6^A‐specific antibody followed by RNA sequencing (MeRIP‐seq) was performed. MeRIP‐seq identified 8992 and 8395 m^6^A peaks in the control and METTL3 knockdown BEAS‐2B cells, respectively (fold change > 2) (Figure 5B). The m^6^A consensus sequence GGAC motif was highly enriched within m^6^A sites in both the control and METTL3 knockdown cells (Figure 5C). Moreover, m^6^A peaks were abundant in the vicinity of start and stop codons (Figure 5D). The m^6^A distribution pattern was similar between the two groups (Figure 5E). Kyoto Encyclopedia of Genes and Genomes (KEGG) pathway enrichment analysis demonstrated that a few the hypomethylated m^6^A transcripts were associated with the mammalian target of rapamycin (mTOR), insulin and adenosine 5'‐monophosphate (AMP)‐activated protein kinase (AMPK) signaling pathways (Figure 5F). In addition, the Gene ontology (GO) analysis showed that some hypomethylated m^6^A transcripts were functionally related to the regulation of catalytic activity, insulin reporter signaling pathway and regulation of the cellular response to insulin stimulus (Figure S4C, Supporting Information).

To further investigate the potential targets involved in m^6^A‐regulated EMT in BEAS‐2B cells, 7 candidate genes (FOXO1, SEMA6B, PRKAA1, PRKCA, CREB3, AKT1S1 and ZNF469) were screened among the hypomethylated m^6^A transcripts. To some extent, all of these candidates were functionally associated with IR‐responsive properties, EMT and m^6^A methylation, which was consistent with the results of the GO and KEGG analyses. Then, the impact of METTL3 on the 7 candidate genes was evaluated by qRT‐PCR and Western blot assay. As a result, FOXO1 was selected for further confirmation, because of its significant upregulation following METTL3 silencing after IR (Figure 5G) and its involvement in cell metabolism, insulin resistance and oxidative stress resistance.^[^ ^21^ ^]^ In contrast, the other 6 candidate genes showed almost no variations in METTL3 knockdown cells after IR ( Figure S4D–J, Supporting Information). Consistent with this finding, the inactivation of METTL3 elevated the protein level of FOXO1 in response to IR both in vitro (Figure 5H) and in vivo (Figure 5I) and vice versa (Figure S4K, Supporting Information). Therefore, FOXO1 was identified as a potential target of METTL3‐mediated m^6^A modification for further investigation.

Next, a MeRIP‐qPCR assay was utilized to confirm the finding that METTL3 targets FOXO1 mRNA for m^6^A modification.^[^ ^22^ ^]^ As expected, a marked reduction in the FOXO1 mRNA m^6^A level following METTL3 silencing was confirmed by MeRIP‐qPCR (Figure 5J). A m^6^A peak was detected in the 3’UTR of FOXO1 mRNA (chr13:41131781‐41132160) and was diminished by METTL3 depletion (Figure S4L, Supporting Information). Furthermore, to determine the effect of m^6^A modification on FOXO1 expression, a luciferase reporter vector containing the wild‐type (WT) or mutated FOXO1 3’UTR in which the adenosine bases in the m^6^A consensus sequences were replaced by cytosine, was constructed (Figure 5K). The forced expression of WT FOXO1, but not mutated FOXO1, substantially increased luciferase activity in the absence of METTL3 expression (Figure 5L). Reciprocally, METTL3 overexpression suppressed the luciferase activity driven by WT FOXO1 but not that driven by the mutated FOXO1‐fused reporter (Figure S4M, Supporting Information). Collectively, these results implied that the regulation of FOXO1 is controlled by METTL3‐mediated m^6^A modification.

### 2.5 Knockdown of METTL3 Potentiates FOXO1 mRNA Stability in a YTHDF2‐Dependent Manner

**Figure 6:**
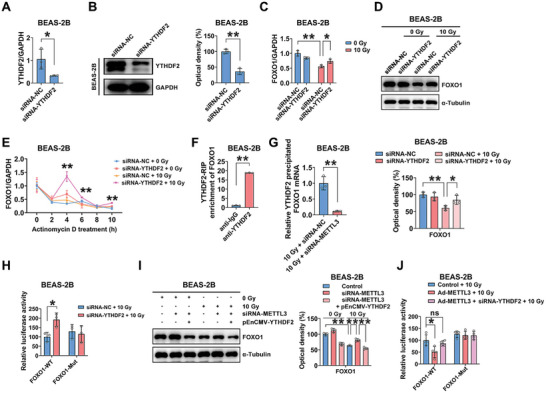
Silencing METTL3 increases FOXO1 mRNA stability through a YTHDF2‐dependent mechanism. A,B) YTHDF2 mRNA and protein levels in control and YTHDF2 knockdown BEAS‐2B cells (*n* = 3; mean ± SD; *p < 0.05, **p < 0.01; Student's t‐test). BEAS‐2B cells were transfected with or without siRNA‐YTHDF2 prior to exposure to 0 or 10 Gy X‐irradiation. The C) mRNA and D) protein levels of FOXO1 were measured (*n* = 3; mean ± SD; *p < 0.05, **p < 0.01; Student's t‐test). E) FOXO1 mRNA stability was analyzed in BEAS‐2B cells treated with actinomycin D for 2, 4, 6, 8, and 10 h (normalized to 0 h) (*n* = 3; mean ± SD; **p < 0.01; Student's t‐test). F) RIP was performed in BEAS‐2B cells with anti‐YTHDF2 antibody prior to qRT‐PCR analysis to measure the amount of FOXO1 mRNA (*n* = 3; mean ± SD; **p < 0.01; Student's t‐test). G) BEAS‐2B cells were transfected with or without siRNA‐METTL3 prior to exposure to 10 Gy X‐irradiation. RIP was performed with an anti‐YTHDF2 antibody prior to qRT‐PCR analysis with primers specific for FOXO1 mRNA (*n* = 3; mean ± SD; **p < 0.01; Student's t‐test). H) The relative activity of the WT and mutated luciferase reporters was determined in YTHDF2‐depleted BEAS‐2B cells exposed to 10 Gy X‐irradiation (*n* = 3; mean ± SD; *p < 0.05; Student's t‐test). I) Western blot and quantitative analyses of FOXO1 expression in BEAS‐2B cells transfected with siRNA‐METTL3 and/or the YTHDF2 overexpression vector (*n* = 3; mean ± SD; **p < 0.01; Student's t‐test). J) Luciferase vectors with the WT or mutated FOXO1 3’UTR were transfected into BEAS‐2B cells with or without METTL3 overexpression or combined METTL3 overexpression and YTHDF2 knockdown prior to irradiation. Relative luciferase activity was determined (*n* = 4; mean ± SD; *p < 0.05, ns: not significant; Student's t‐test).

The m^6^A readers bind m^6^A‐modified mRNAs for the disposition of target RNAs.^[^ ^23^ ^]^ The results above showed that the mRNA level of FOXO1 might be modified by METTL3. Next, the possible mechanism involved was investigated. The m^6^A modification‐mediated mRNA degradation is regulated by YTHDF1‐3,^[^ ^23^, ^24^ ^]^ among which YTHDF2 is a major decay‐inducing reader protein.^[^ ^24^ ^]^ Therefore, we explored the effect of YTHDF1‐3 on FOXO1 mRNA stability. As expected, increased FOXO1 mRNA and protein expression was detected in YTHDF2 knockdown BEAS‐2B cells after IR (**Figure** **6**A–D), while YTHDF1/3 had a limited effect on FOXO1 expression in BEAS‐2B cells (Figure S4N–S, Supporting Information). Notably, striking increases in FOXO1 mRNA stability were observed in YTHDF2 knockdown BEAS‐2B cells after 10 Gy X‐irradiation (Figure 6E). As shown in Figure 6F, YTHDF2 was enriched on FOXO1 mRNA, while METTL3 knockdown significantly reduced the amount of YTHDF2 bound to FOXO1 mRNA (Figure 6G). These results confirmed that FOXO1 is a target of YTHDF2. In addition, YTHDF2 silencing increased the luciferase activity driven by the 3’UTR fragment of m^6^A‐modified WT FOXO1, whereas the increase in luciferase activity was abrogated by FOXO1 mutation (Figure 6H).

In contrast, YTHDF2 overexpression reversed the increase in FOXO1 protein expression in METTL3‐deficient BEAS‐2B cells after IR exposure (Figure 6I). Conversely, YTHDF2 knockdown moderately rescued the METTL3‐mediated suppression of FOXO1 expression in irradiated BEAS‐2B cells (Figure S4T, Supporting Information). Consistent with this finding, METTL3 overexpression inhibited the luciferase activity driven by the 3’UTR fragment of WT FOXO1, and luciferase activity was rescued by YTHDF2 knockdown (Figure 6J). However, neither METTL3 upregulation nor YTHDF2 downregulation affected the luciferase activity driven by mutated FOXO1 (Figure 6J). Taken together, these data indicated that METTL3‐mediated m^6^A modification suppresses FOXO1 expression in a YTHDF2‐dependent manner.

### 2.6 AKT and ERK Signaling Pathways Are Involved in METTL3‐Mediated Regulation of FOXO1 Expression during IR‐Induced EMT

**Figure 7:**
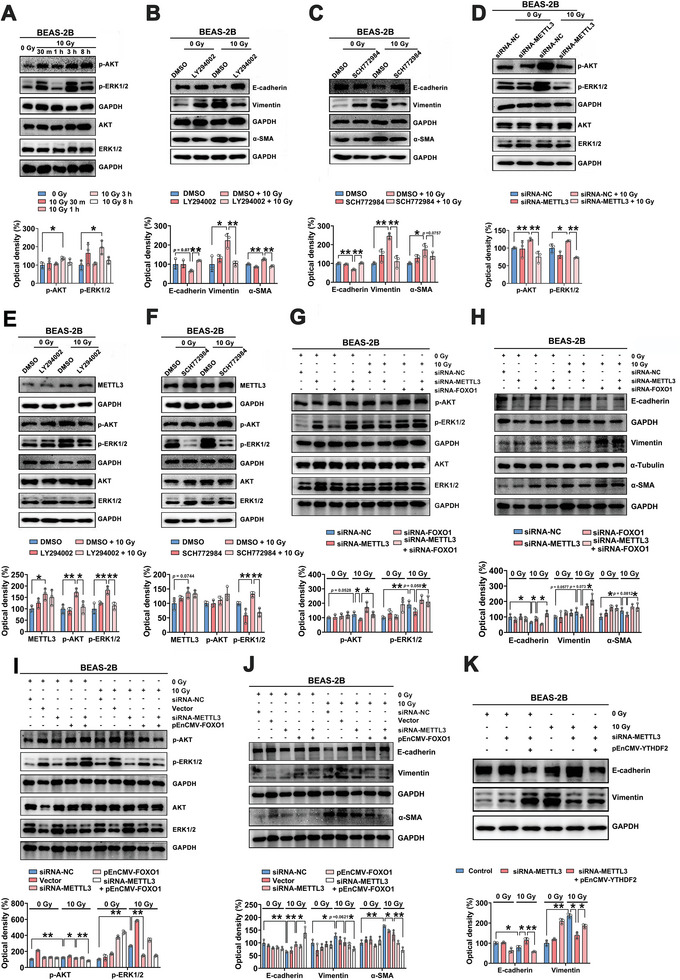
METTL3/FOXO1‐dependent AKT‐ERK activation is critical for IR‐induced EMT. A) Western blot and quantitative analyses of AKT, p‐AKT, ERK and p‐ERK in BEAS‐2B cells at different times after 0 or 10 Gy irradiation (*n* = 3; mean ± SD; *p < 0.05; Student's t‐test). B) BEAS‐2B cells were incubated with or without the selective AKT inhibitor LY294002 (1 × 10^‐6^ m) for 2 h before irradiation with 10 Gy. Western blot and quantitative analyses of E‐cadherin, Vimentin, and *α*‐SMA expression at 72 h post‐irradiation (*n* = 3; mean ± SD; *p < 0.05, **p < 0.01; Student's t‐test). C) BEAS‐2B cells were incubated with or without SCH772984 (0.5 × 10^‐6^ m) for 2 h before irradiation. Cell lysates were collected, and the protein levels of EMT‐related markers at 72 h post‐irradiation were measured by a Western blot analysis followed by quantitative analyses using Image J software (*n* = 3; mean ± SD; *p < 0.05, **p < 0.01; Student's t‐test). D) Protein levels of AKT, ERK, p‐AKT and p‐ERK in METTL3 knockdown BEAS‐2B cells treated with or without 10 Gy irradiation (*n* = 3; mean ± SD; *p < 0.05, **p < 0.01; Student's t‐test). E,F) BEAS‐2B cells were incubated with or without the AKT or ERK inhibitor for 2 h prior to 10 Gy irradiation. The protein levels of METTL3, AKT, ERK, p‐AKT and p‐ERK were measured (*n* = 3; mean ± SD; *p < 0.05, **p < 0.01; Student's t‐test). G) The protein levels of AKT, ERK, p‐AKT and p‐ERK in BEAS‐2B cells transfected with siRNA‐METTL3 and/or siRNA‐FOXO1 and treated with or without irradiation (*n* = 3; mean ± SD; *p < 0.05, **p < 0.01; Student's t‐test). H) EMT‐related protein levels in BEAS‐2B cells transfected with siRNA‐METTL3 and/or siRNA‐FOXO1 and treated with or without irradiation (*n* = 3; mean ± SD; *p < 0.05; Student's t‐test). I) The protein levels of AKT, ERK, p‐AKT and p‐ERK were measured in BEAS‐2B cells transfected with siRNA‐METTL3 and/or the FOXO1 overexpression vector and treated with or without irradiation (*n* = 3; mean ± SD; *p < 0.05, **p < 0.01; Student's t‐test). J) EMT‐related protein levels were measured in BEAS‐2B cells transfected with siRNA‐METTL3 and/or the FOXO1 overexpression vector and treated with or without irradiation (*n* = 3; mean ± SD; *p < 0.05, **p < 0.01; Student's t‐test). K) Western blot and quantitative analyses of E‐cadherin and Vimentin expression in BEAS‐2B cells treated with siRNA‐METTL3 and/or the YTHDF2 overexpression vector (*n* = 3; mean ± SD; *p < 0.05, **p < 0.01; Student's t‐test).

The AKT and ERK signaling pathways play critical roles in mediating EMT.^[^ ^25^ ^]^ Therefore, we sought to determine whether and how AKT and ERK signaling were involved in IR‐induced EMT. It was demonstrated that the levels of phosphorylated AKT and ERK were significantly increased after exposure to 10 Gy irradiation, particularly at 3 h post‐irradiation (**Figure** **7**A; Figure S5A, Supporting Information). By applying LY294002, a selective AKT inhibitor, we determined that AKT could mediate IR‐induced EMT. LY294002 markedly altered the protein levels of E‐cadherin, VE‐cadherin, Vimentin, and *α*‐SMA at 72 h after irradiation, indicating that blocking AKT could inhibit IR‐induced EMT (Figure 7B; Figure S5B, Supporting Information). In addition, the ERK‐selective inhibitor SCH772984 markedly reversed the EMT phenotype in vitro (Figure 7C; Figure S5C, Supporting Information).

Since METTL3 may affect the AKT and ERK pathways,^[^ ^26^ ^]^ we investigated the possible regulatory relationships between METTL3 and AKT/ERK. METTL3 knockdown inhibited the IR‐induced activation of AKT and ERK at 3 h post‐IR in both BEAS‐2B cells and HUVECs, but the total expression levels of AKT and ERK were not significantly altered (Figure 7D; Figure S5D, Supporting Information), which was further verified in lung tissues from mice with RILI (Figure S5E, Supporting Information). In contrast, the AKT inhibitor had a limited influence on the protein expression of METTL3, even after IR exposure (Figure 7E; Figure S5F, Supporting Information). Similarly, blocking ERK with SCH772984 did not affect METTL3 protein expression (Figure 7F; Figure S5G, Supporting Information). The above results indicated that AKT and ERK might be downstream effectors of METTL3 and that METTL3 may promote IR‐induced EMT at least partially through the AKT and ERK signaling pathways.

In addition, the relationship between EMT and the AKT and ERK signaling pathways was identified. The level of phosphorylated ERK was sharply decreased in irradiated pulmonary cells pretreated with LY294002, although the total ERK expression level was unchanged (Figure 7E; Figure S5F, Supporting Information), suggesting that AKT blockade could overcome IR‐induced ERK activation. Moreover, we speculated whether inhibiting ERK can attenuate IR‐induced AKT activation, and we found that SCH772984 did not significantly affect IR‐induced AKT phosphorylation (Figure 7F; Figure S5G, Supporting Information). Despite the intricacies of the AKT and ERK signaling pathways, this study suggested that AKT may be the upstream effector of ERK in IR‐induced EMT in vitro.

Furthermore, we explored whether the METTL3/FOXO1 axis is associated with AKT and ERK in the regulation of IR‐induced EMT. BEAS‐2B cells were transfected with siRNA‐METTL3 and/or siRNA‐FOXO1, and FOXO1 inhibition was found to partially reverse the decreases in the p‐AKT and p‐ERK levels in METTL3‐deficient cells at 3 h post‐irradiation (Figure 7G). In addition, FOXO1 depletion partially reduced the inhibitory effect of METTL3 knockdown on IR‐induced EMT (Figure 7H; Figure S5H, Supporting Information). In contrast, FOXO1 overexpression promoted the siRNA‐METTL3‐induced inhibition of AKT phosphorylation and EMT in response to IR exposure (Figure 7I,J; Figure S5I, Supporting Information). Moreover, in irradiated BEAS‐2B cells, the upregulation of FOXO1 counteracted the increases in AKT and ERK phosphorylation at 3 h postirradiation (Figure S5J, Supporting Information) and the acceleration of IR‐induced EMT (Figure S5K,L, Supporting Information) induced by METTL3 overexpression. In addition, METTL3 depletion‐elicited inhibition of IR‐induced EMT could be overcome by overexpressing YTHDF2 (Figure 7K). Conversely, YTHDF2 silencing reversed the decrease in the E‐cadherin protein levels and the increase in Vimentin expression in METTL3‐overexpressing BEAS‐2B cells exposed to IR (Figure S5M, Supporting Information). Taken together, the results above implied that the METTL3/FOXO1 axis participates in IR‐induced EMT via AKT and ERK signaling.

## 3 Discussion

The m^6^A modification has been shown to participate in the regulation of biological functions and in the progression of multiple human diseases.^[^ ^14^, ^15^, ^16^, ^24^, ^27^ ^]^ In the present study, we showed that m^6^A modification is positively correlated with IR‐induced EMT, which modulated the development and progression of RILI. In brief, IR induced significant upregulation of m^6^A modification and EMT during RILI both in vivo and in vitro. The suppression of m^6^A modification by METTL3 knockdown obviously alleviated IR‐induced EMT both in vivo and in vitro. The interaction between METTL3 and FOXO1 was verified by MeRIP‐seq and luciferase reporter assays. Fundamentally, our results indicated that METTL3‐mediated m^6^A modification of FOXO1 mRNA recruits YTHDF2 and downregulates it expression, which is followed by activation of the AKT and ERK signaling pathways.

Accumulating evidence has revealed the key role of m^6^A modification in regulating tumor proliferation and development in several cancers, such as gastric cancer and esophageal cancer.^[^ ^15^, ^24^ ^]^ Alterations in m^6^A modifications may be mediated by the regulation of DNA damage repair, downstream adaptive responses, and the tumor microenvironment to protect tumor cells from drug‐mediated cell death.^[^ ^28^ ^]^ In addition, m^6^A modification is involved in organ injuries and fibrosis. For example, genetic and pharmacological inhibition of METTL3 alleviated renal inflammation and injury both in vitro and in vivo.^[^ ^29^ ^]^ METTL3‐mediated m^6^A modification was found to play a profibrotic role in the postinfarct myocardium, and silencing METTL3 mitigated collagen production both in vitro and in vivo.^[^ ^30^ ^]^ Notably, an intervention targeting METTL3‐mediated m^6^A modification inhibited the fibroblast‐to‐myofibroblast transition (FMT) in pulmonary fibrosis.^[^ ^16^ ^]^ Here, we revealed a novel role of m^6^A modification in regulating RILI. An elevated m^6^A level during RILI was demonstrated and found to be closely related to IR‐induced EMT, which might be caused by METTL3 upregulation and ALKBH5 downregulation. Next, by applying a loss/gain‐of‐function approach in cell and mouse models of RILI, we confirmed that METTL3 and ALKBH5 are crucial regulators of m^6^A modification in the regulation of IR‐induced EMT during RILI. Previous studies suggested that m^6^A modification can either promote or inhibit cell proliferation in different scenarios.^[^ ^15^, ^16^ ^]^ Here, we demonstrated that either METTL3 deletion or ALKBH5 overexpression attenuates EMT and confers protection against IR‐induced damage both in vitro and in vivo. This finding indicated the potential role of m^6^A modification in the response to radiation.

Considering the importance of m^6^A modification in IR‐induced EMT, we further delineated the underlying mechanism by MeRIP‐seq, a widely accepted method for transcriptome‐wide screening of m^6^A localization.^[^ ^11^ ^]^ Subsequently, FOXO1 was identified as a candidate target of METTL3‐mediated m^6^A modification during IR‐induced EMT. The *FOXO1* gene belongs to a family of transcription factors characterized by a conserved forkhead domain. First identified in chromosomal translocations found in human tumors,^[^ ^31^ ^]^ FOXO1 was thought to play roles in the occurrence and development of several human cancers.^[^ ^32^ ^]^ In addition, FOXO1 has also been considered to play critical roles in energy metabolism, cell proliferation and oxidative stress resistance.^[^ ^21^ ^]^ The present study showed that FOXO1 is a key target of METTL3‐mediated m^6^A modification during IR‐induced EMT. First, MeRIP‐qPCR was utilized to confirm the finding that METTL3 targets FOXO1 mRNA for m^6^A modification. Second, FOXO1 expression was decreased during IR‐induced EMT, and this decrease was abrogated by METTL3 silencing. Additionally, a luciferase reporter vector containing the WT or mutated FOXO1 3’UTR was constructed to confirm that FOXO1 is involved in METTL3‐mediated m^6^A modification.

The biological functions of m^6^A modification depend on reader proteins. YTHDF1‐3 destabilize m^6^A‐modified RNAs;^[^ ^23^, ^24^ ^]^ of these, YTHDF2 is a major degradation‐inducing reader protein.^[^ ^24^ ^]^ For example, YTHDF2 promoted liver cancer progression by promoting the degradation of suppressor of cytokine signaling 2 (SOCS2).^[^ ^33^ ^]^ Consistent with the abovementioned studies, our data indicated that METTL3‐mediated m^6^A modification suppresses FOXO1 expression in a YTHDF2‐dependent manner. By applying RIP, a method used to detect the association between individual proteins and specific RNAs,^[^ ^34^ ^]^ we demonstrated that YTHDF2 is enriched on FOXO1 mRNA. Moreover, YTHDF2 knockdown was found to increase the expression of FOXO1 in irradiated BEAS‐2B cells, while YTHDF1/3 had a limited effect on FOXO1 expression. Collectively, these items of evidence supported the hypothesis that the FOXO1 transcript is a direct target of YTHDF2.

Furthermore, a mechanistic study was carried out to elucidate the mechanism by which FOXO1 regulates IR‐induced EMT. First, the AKT‐ERK signaling pathway was found to be associated with the METTL3‐mediated promotion of EMT after irradiation. FOXO1 is generally accepted as a downstream regulator of AKT.^[^ ^35^ ^]^ However, recent studies have shown that FOXO1 also acts as an upstream factor of AKT in gastric cancer and activates this pathway to mediate cisplatin resistance.^[^ ^36^ ^]^ In this work, FOXO1 was shown to activate the AKT pathway, which was consistent with the findings reported by Zhao et al. in nasopharyngeal carcinoma.^[^ ^37^ ^]^ FOXO1 target genes reportedly greatly differ in different cell types,^[^ ^38^ ^]^ which may at least partially account for these discrepancies. In addition, ERK has been reported to be regulated by FOXO1 activation,^[^ ^39^ ^]^ which was consistent with our finding that FOXO1 activates the ERK pathway. Previous studies documented that FOXO1 is involved in EMT^[^ ^40^ ^]^ and ameliorates fibrosis in numerous organs.^[^ ^35^, ^41^ ^]^ Consistent with these observations, our results showed that FOXO1 expression is decreased in irradiated pulmonary epithelial cells and lung tissues. Moreover, FOXO1 overexpression and deletion alleviated and enhanced IR‐induced EMT in pulmonary epithelial cells, respectively. As both the AKT and ERK signaling pathways mediated EMT,^[^ ^25^ ^]^ we concluded that FOXO1 participates in IR‐induced EMT by activating the AKT and ERK signaling pathways.

**Figure 8:**
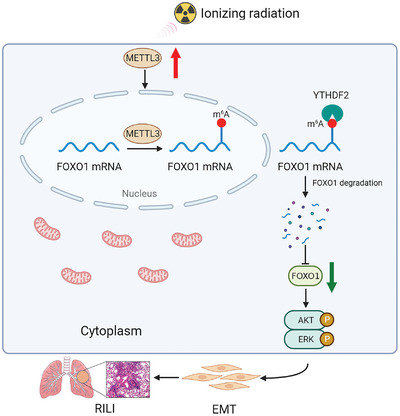
Schematic representation of the METTL3/FOXO1 axis in IR‐induced EMT. The overexpression of METTL3 induced by IR increases the m^6^A level of FOXO1 mRNA, which recruits YTHDF2 to FOXO1 mRNA to downregulate FOXO1 expression. The downregulation of FOXO1 expression in turn regulates the activation of the AKT and ERK pathways, ultimately facilitating the progression of IR‐induced EMT during RILI.

In summary, the present study provides novel insights into the key role of METTL3 in regulating the occurrance and progression of RILI via YTHDF2‐dependent mediation of FOXO1 expression and subsequent activation of the AKT and ERK signaling pathways (**Figure** **8**). However, further in‐depth analyses are critical for revealing the complete METTL3‐associated regulatory network in RILI. First, since no good clinical therapeutic approach is available to prevent RILI, modulating m^6^A modification may provide valuable guidance for the development of novel preventive and therapeutic strategies. The number of specific inhibitors of m^6^A regulators is limited, and screening effective inhibitors against m^6^A modifications merits further investigation. STM2457, a highly specific inhibitor of METTL3, has recently been developed and proven to have an anti‐leukemic effect.^[^ ^42^ ^]^ Therefore, further studies investigating the effects of METTL3 inhibitors in RILI are highly warranted. Second, the biological functions of m^6^A modification require its selective recognition by specific binding proteins.^[^ ^23^ ^]^ Notably, these biological processes may be regulated by multiple m^6^A readers.^[^ ^43^ ^]^ In our study, m^6^A methylation was negatively correlated with FOXO1 expression; thus, we only focused on YTHDF1‐3, and whether other readers regulate FOXO1 may be explored in the future. Third, the specific mechanism by which ALKBH5 regulates EMT is another future research direction. In addition, other candidate genes may also participate in the METTL3‐associated regulatory network, which cannot be ruled out and needs further verification.

## 4 Experimental Section

### Cell Culture and Irradiation

Human pulmonary epithelial cells (BEAS‐2B), human umbilical vein endothelial cells (HUVECs) and murine pulmonary epithelial cells (MLE‐12) were purchased from the American Type Culture Collection (ATCC; Manassas, VA, USA). Cells were maintained in high‐glucose Dulbecco's modified Eagle's medium (HyClone, Logan, UT, USA) supplemented with 10% fetal bovine serum (Gibco, Grand Island, NY, USA) and incubated at 37 °C in a humidified atmosphere with 5% CO_2_. Cells were exposed to IR (0, 2, 5, or 10 Gy) using an X‐ray linear accelerator (RadSource, Suwanee, GA, USA) at a fixed dose rate of 1.15 Gy min^‐1^.

### Quantification of m^6^A Modification

Total RNA from pulmonary cells or lung tissues was extracted using TRIzol reagent (Invitrogen, Carlsbad, CA, USA) according to the manufacturer's instructions. The mRNA was purified from total RNA using the Dynabead mRNA Purification Kit (Invitrogen, Carlsbad, CA, USA). Two hundred nanograms of total RNA or mRNA were measured with a m^6^A RNA Methylation Quantification Kit (Abcam, Cambridge, MA, USA) according to the manufacturer's instructions. In brief, the test wells were coated with 200 ng total RNA or mRNA. Dilutions of the capture antibody solution and detection antibody solution were then added to each test well, and the optical absorbance of the samples was measured at 450 nm.

### m^6^A‐RIP and m^6^A Sequencing (MeRIP‐Seq) Assays

Total polyadenylated RNA was isolated from transfected BEAS‐2B cells exposed to 10 Gy X‐rays at 72 h post‐irradiation using TRIzol reagent. MeRIP‐seq was performed by Cloudseq Biotech, Inc. (Shanghai, China) according to a published procedure with slight modifications.^[^ ^11^ ^]^ Briefly, fragmented mRNA was incubated with an anti‐m^6^A polyclonal antibody in IPP buffer (10 × 10^‐3^ m Tris–HCl, 150 × 10^‐3^ m NaCl, 0.1% NP40, pH 7.4) at 4 °C for 2 h. Both the input sample without immunoprecipitation and the m^6^A IP samples were used for RNA‐seq library generation with a NEBNext Ultra Directional RNA Library Prep Kit (New England BioLabs, Ipswich, MA, USA), and library sequencing was performed on an Illumina NovaSeq 6000 instrument with 150 bp paired‐end reads. The MeRIP‐seq data were analyzed according to previously described protocols.^[^ ^44^ ^]^ The paired‐end reads were quality controlled by Q30, followed by the removal of 3’‐adapters and low‐quality reads by cutadapt software (v1.9.3).^[^ ^11^ ^]^ Then, the clean reads were aligned to the reference genome (UCSC HG19) by HISAT2 software (v2.0.4).^[^ ^45^ ^]^ The peaks were identified by MACS. GO analysis involving CC was performed with a database for annotation, visualization, and integrated discovery. The *p* value denotes the significance of enrichment of the GO term in the genes. Methylated sites on RNAs (peaks) were identified by MACS software.^[^ ^46^ ^]^ Differentially methylated sites were identified by diffReps software.^[^ ^47^ ^]^ The peaks identified by both software programs that overlapped exons of mRNA were identified and chosen with homemade scripts. GO and pathway enrichment analyses of differentially methylated protein‐coding genes were performed.

### MeRIP Quantitative PCR (MeRIP‐qPCR)

MeRIP‐qPCR was conducted according to a published protocol.^[^ ^48^ ^]^ In brief, a Magna MeRIP m^6^A kit (#17‐10499, Millipore, Bedford, MA, USA) was used according to the manufacturer's instructions. m^6^A enrichment was analyzed by qRT‒PCR with specific primers, and the data were normalized to the input. The primer sequences were as follows:

FOXO1 forward: 5’‐TTCCTCTAAGCACAGCCTCC‐3’;
FOXO1 reverse: 5’‐TAATGGCACGGGAGGAAAGT‐3’.

### RNA Immunoprecipitation (RIP)

RIP assays of BEAS‐2B cells were performed as previously described.^[^ ^49^ ^]^ Briefly, a Magna RIP Kit (#17‐700, Millipore, Bedford, MA, USA) was adopted, and enough cell lysates were incubated with magnetic beads coated with 5 µg of anti‐YTHDF2 antibody or 5 µg of rabbit IgG at 4 °C overnight. After washing, the lysates were digested with Proteinase K, and RNA bound to the immunoprecipitated proteins was then purified and analyzed by qRT‒PCR.

### RNA Decay Assay

To measure RNA stability, the cells were transfected with YTHDF2‐siRNA, followed by treatment with actinomycin D at 5 µg mL^‐1^. The cells were collected after 0, 2, 4, 6, 8 and 10 h. Total RNA was isolated and subjected to qRT‒PCR to quantify the relative abundance of FOXO1 mRNA.^[^ ^44^ ^]^

### RILI Animal Models

Animal experimental protocols were approved by the Animal Experimentation Ethics Committee at Soochow University (No. SUDA20220816A01, SUDA20230208A02 and SUDA20230208A03, Suzhou, China). Male SD rats (250‐300 g) were purchased from Shanghai SLAC Laboratory Animal Co., Ltd. (Shanghai, China). A model of RILI induced by unilateral pulmonary irradiation was established in the rats as previously reported.^[^ ^17^ ^]^ The right lung of each rat was irradiated with 6 MeV X‐rays (Varian 23EX linear accelerator, Palo Alto CA) at a single dose of 0 or 20 Gy (*n* = 25), and the dose rate was 5 Gy min^‐1^. The left chest and other parts of the body were protected with 3 mm of lead. Correct positioning of the fields was controlled for each rat using a therapy simulator (Huestis Cascade Simulator, Bristol, RI) based on previous reports.^[^ ^17^ ^]^ The rats were sacrificed, and samples were obtained at 3, 7, 12, 18 and 26 weeks after irradiation.

Male C57BL/6 mice aged 6–8 weeks were purchased from Shanghai SLAC Laboratory Animal Co., Ltd. (Shanghai, China). The mice were housed under a 12 h light/dark cycle and had free access to food and water. In total, 60 mice were randomly divided into the following three groups: 1) a noninstrumented control group (*n* = 20); 2) an irradiation group injected with Ad‐NC (*n* = 20); and 3) an irradiation group injected with Ad‐ALKBH5 (*n* = 20). A single dose of 20 Gy irradiation was precisely administered to the right lung area with an aperture of 10 mm in the mice in the irradiation groups using a small animal radiotherapy simulation localization machine (X‐RAD SmART, Precision X‐Ray Inc., Branford, CT) after positioning. The mice in the irradiation groups separately received tail intravenous injection of 100 µL of Ad‐NC (1.0 × 10^10^ pfu mL^‐1^) or Ad‐ALKBH5 (1.0 × 10^10^ pfu mL^‐1^). In vivo infection was performed one day before irradiation and ten days after irradiation. The mice were observed and weighed weekly to ensure that the interventions were well tolerated. The mice were sacrificed, and peripheral blood and tissue samples were collected at 1, 4, 8 and 16 weeks after irradiation.

### Statistical Analysis

The data are expressed as the mean ± SD of at least three independent experiments. The differences among multiple treatment groups were analyzed by one‐way ANOVA, followed by Tukey's test for multiple comparisons. Student's *t* test was used to analyze the differences between two groups to determine statistical significance. The statistical analyses were performed using Prism 8 software (GraphPad Software, La Jolla, CA, USA). Differences with *P* < 0.05 were considered significant.

The other methods are described in detail in the Materials and Methods, Supporting Information.

## Conflict of Interest

The authors declare no conflict of interest.

## Author Contributions

Y.J. and L.W. conceived and designed the study. Y.F. carried out the molecular biology studies. Y.J. drafted the manuscript and the figures. P.Y., H.G., L.G., Z.Y. and J.W. performed the animal experiments. W.Z. and Q.Z. performed the statistical analysis. Y.J. and J.C. modified the manuscript. All authors read and approved the final manuscript.

## Supporting information

Supporting Information

## Data Availability

The data that support the findings of this study are available from the corresponding author upon reasonable request.

## References

[1] D. E. Citrin , N. Engl. J. Med. 2017, 377, 1065. 28902591 10.1056/NEJMra1608986

[2] a) L. Giuranno , J. Ient , D. De Ruysscher , M. A. Vooijs , Front. Oncol. 2019, 9, 877; 31555602 10.3389/fonc.2019.00877 PMC6743286

[3] a) J. Mahmood , S. Jelveh , V. Calveley , A. Zaidi , S. R. Doctrow , R. P. Hill , Radiat. Res. 2011, 176, 770; 22013884 10.1667/rr2562.1 PMC3252890

[4] M. Yi , B. Liu , Y. Tang , F. Li , W. Qin , X. Yuan , Biomed Res. Int. 2018, 2018, 4135806. 29619372 10.1155/2018/4135806 PMC5830288

[5] a) Y.‐T. Oh , O. K. Noh , H. Jang , M. Chun , K. J. Park , K. J. Park , M.‐H. Kim , H.‐J. Park , Radiother. Oncol. 2012, 102, 343; 22342420 10.1016/j.radonc.2012.02.003

[6] R. C. Stone , I. Pastar , N. Ojeh , V. Chen , S. Liu , K. I. Garzon , M. Tomic‐Canic , Cell Tissue Res. 2016, 365, 495. 27461257 10.1007/s00441-016-2464-0 PMC5011038

[7] S. Lamouille , J. Xu , R. Derynck , Nat. Rev. Mol. Cell Biol. 2014, 15, 178. 24556840 10.1038/nrm3758 PMC4240281

[8] D. Y. Shu , E. Butcher , M. Saint‐Geniez , Int. J. Mol. Sci. 2020, 21, 4271. 32560057 10.3390/ijms21124271 PMC7349630

[9] a) T. A. Wynn , J. Pathol. 2008, 214, 199; 18161745 10.1002/path.2277 PMC2693329

[10] a) K. C. Flanders , Int. J. Exp. Pathol. 2004, 85, 47; 15154911 10.1111/j.0959-9673.2004.00377.x PMC2517464

[11] K. D. Meyer , Y. Saletore , P. Zumbo , O. Elemento , C. E. Mason , S. R. Jaffrey , Cell 2012, 149, 1635. 22608085 10.1016/j.cell.2012.05.003 PMC3383396

[12] a) S. Oerum , V. Meynier , M. Catala , C. Tisné , Nucleic Acids Res. 2021, 49, 7239; 34023900 10.1093/nar/gkab378 PMC8287941

[13] a) X. Deng , R. Su , H. Weng , H. Huang , Z. Li , J. Chen , Cell Res. 2018, 28, 507; 29686311 10.1038/s41422-018-0034-6 PMC5951805

[14] a) Z. Xu , K. Jia , H. Wang , F. Gao , S. Zhao , F. Li , J. Hao , Cell Death Dis. 2021, 12, 32; 33414476 10.1038/s41419-020-03312-0 PMC7791055

[15] a) X. Chen , M. Xu , X. Xu , K. Zeng , X. Liu , B. Pan , C. Li , L. Sun , J. Qin , T. Xu , B. He , Y. Pan , H. Sun , S. Wang , Mol. Cancer 2020, 19, 106; 32552762 10.1186/s12943-020-01220-7 PMC7298962

[16] a) B. Han , C. Chu , X. Su , N. Zhang , L. Zhou , M. Zhang , S. Yang , L. Shi , B. Zhao , Y. Niu , R. Zhang , Nanotoxicology 2020, 14, 1; 31502903 10.1080/17435390.2019.1661041

[17] L. Xie , J. Zhou , S. Zhang , Q. Chen , R. Lai , W. Ding , C. Song , X. Meng , J. Wu , Radiat. Oncol. 2014, 9, 111. 24886372 10.1186/1748-717X-9-111 PMC4044330

[18] X. Lei , N. He , L. Zhu , M. Zhou , K. Zhang , C. Wang , H. Huang , S. Chen , Y. Li , Q. Liu , Z. Han , Z. Guo , Z. Han , Z. Li , Antioxid. Redox Signaling 2021, 35, 849. 10.1089/ars.2019.7965 32664737

[19] D. Hu , Y. Zhang , R. Cao , Y. Hao , X. Yang , T. Tian , J. Zhang , Transl. Lung Cancer Res. 2020, 9, 2440. 33489805 10.21037/tlcr-20-1272 PMC7815363

[20] X. Tian , F. Wang , Y. Luo , S. Ma , N. Zhang , Y. Sun , C. You , G. Tang , S. Li , Y. Gong , C. Xie , Front. Oncol. 2018, 8, 542. 30533397 10.3389/fonc.2018.00542 PMC6265406

[21] a) D. G. Sedding , Biol. Chem. 2008, 389, 279; 18208360 10.1515/BC.2008.033

[22] D. Dominissini , S. Moshitch‐Moshkovitz , S. Schwartz , M. Salmon‐Divon , L. Ungar , S. Osenberg , K. Cesarkas , J. Jacob‐Hirsch , N. Amariglio , M. Kupiec , R. Sorek , G. Rechavi , Nature 2012, 485, 201. 22575960 10.1038/nature11112

[23] a) C. D. Allis , T. Jenuwein , Nat. Rev. Genet. 2016, 17, 487; 27346641 10.1038/nrg.2016.59

[24] a) Y. Lee , J. Choe , O. H. Park , Y. K. Kim , Trends Genet. 2020, 36, 177; 31964509 10.1016/j.tig.2019.12.007

[25] X. Liu , L. Sun , S. Zhang , S. Zhang , W. Li , J. Cell. Physiol. 2020, 235, 7747. 31681988 10.1002/jcp.29381

[26] Y. Zhu , X. Pan , N. Du , K. Li , Y. Hu , L. Wang , J. Zhang , Y. Liu , L. Zuo , X. Meng , C. Hu , X. Wu , J. Jin , W. Wu , X. Chen , F. Wu , Y. Huang , FASEB J. 2020, 34, 14371. 32949431 10.1096/fj.202001337R

[27] X. Yang , S. Zhang , C. He , P. Xue , L. Zhang , Z. He , L. Zang , B. Feng , J. Sun , M. Zheng , Mol. Cancer 2020, 19, 46. 32111213 10.1186/s12943-020-1146-4 PMC7047419

[28] B. Li , J. Jiang , Y. G. Assaraf , H. Xiao , Z.‐S. Chen , C. Huang , Drug Resistance Updates 2020, 53, 100720. 32892147 10.1016/j.drup.2020.100720

[29] J.‐N. Wang , F. Wang , J. Ke , Z. Li , C.‐H. Xu , Q. Yang , X. Chen , X.‐Y. He , Y. He , X.‐G. Suo , C. Li , J.‐T. Yu , L. Jiang , W.‐J. Ni , J. Jin , M.‐M. Liu , W. Shao , C. Yang , Q. Gong , H.‐Y. Chen , J. Li , Y.‐G. Wu , X.‐M. Meng , Sci. Transl. Med. 2022, 14, eabk2709. 35417191 10.1126/scitranslmed.abk2709

[30] T. Li , Y. Zhuang , W. Yang , Y. Xie , W. Shang , S. Su , X. Dong , J. Wu , W. Jiang , Y. Zhou , Y. Li , X. Zhou , M. Zhang , Y. Lu , Z. Pan , FASEB J. 2021, 35, e21162. 33150686 10.1096/fj.201903169R

[31] N. Galili , R. J. Davis , W. J. Fredericks , S. Mukhopadhyay , F. J. Rauscher , B. S. Emanuel , G. Rovera , F. G. Barr , Nat. Genet. 1993, 5, 230. 8275086 10.1038/ng1193-230

[32] M. Katoh , M. Katoh , Int. J. Oncol. 2004, 25, 1495. 15492844

[33] M. Chen , L. Wei , C.‐T. Law , F. H.‐C. Tsang , J. Shen , C. L.‐H. Cheng , L.‐H. Tsang , D. W.‐H. Ho , D. K.‐C. Chiu , J. M.‐F. Lee , C. C.‐L. Wong , I. O.‐L. Ng , C.‐M. Wong , Hepatology 2018, 67, 2254. 29171881 10.1002/hep.29683

[34] M. Gagliardi , M. R. Matarazzo , Methods Mol. Biol. 2016, 1480, 73. 27659976 10.1007/978-1-4939-6380-5_7

[35] Z. Xin , Z. Ma , W. Hu , S. Jiang , Z. Yang , T. Li , F. Chen , G. Jia , Y. Yang , Ageing Res. Rev. 2018, 41, 42. 29138094 10.1016/j.arr.2017.11.002

[36] J. Park , Y. S. Ko , J. Yoon , M. A. Kim , J.‐W. Park , W. H. Kim , Y. Choi , J. H. Kim , Y. Cheon , B. L. Lee , Gastric Cancer 2014, 17, 423. 24202965 10.1007/s10120-013-0314-2

[37] M. Zhao , R. Luo , Y. Liu , L. Gao , Z. Fu , Q. Fu , X. Luo , Y. Chen , X. Deng , Z. Liang , X. Li , C. Cheng , Z. Liu , W. Fang , Nat. Commun. 2016, 7, 11309. 27095304 10.1038/ncomms11309 PMC4842991

[38] J.‐H. Paik , R. Kollipara , G. Chu , H. Ji , Y. Xiao , Z. Ding , L. Miao , Z. Tothova , J. W. Horner , D. R. Carrasco , S. Jiang , D. G. Gilliland , L. Chin , W. H. Wong , D. H. Castrillon , R. A. DePinho , Cell 2007, 128, 309. 17254969 10.1016/j.cell.2006.12.029 PMC1855089

[39] a) C.‐W. Pan , X. Jin , Y. Zhao , Y. Pan , J. Yang , R. J. Karnes , J. Zhang , L. Wang , H. Huang , EMBO J. 2017, 36, 995; 28279977 10.15252/embj.201695534 PMC5391142

[40] a) M. Du , Q. Wang , W. Li , X. Ma , L. Wu , F. Guo , S. Zhao , F. Huang , H. Wang , G. Qin , Biochem. Biophys. Res. Commun. 2016, 471, 416; 26902117 10.1016/j.bbrc.2016.02.066

[41] L. Ji , Q. Wang , F. Huang , T. An , F. Guo , Y. Zhao , Y. Liu , Y. He , Y. Song , G. Qin , Oxid. Med. Cell. Longevity 2019, 2019, 3286928. 10.1155/2019/3286928 PMC6431359 30962862

[42] E. Yankova , W. Blackaby , M. Albertella , J. Rak , E. De Braekeleer , G. Tsagkogeorga , E. S. Pilka , D. Aspris , D. Leggate , A. G. Hendrick , N. A. Webster , B. Andrews , R. Fosbeary , P. Guest , N. Irigoyen , M. Eleftheriou , M. Gozdecka , J. M. L. Dias , A. J. Bannister , B. Vick , I. Jeremias , G. S. Vassiliou , O. Rausch , K. Tzelepis , T. Kouzarides , Nature 2021, 593, 597. 33902106 10.1038/s41586-021-03536-w PMC7613134

[43] R. Wu , Y. Liu , Y. Zhao , Z. Bi , Y. Yao , Q. Liu , F. Wang , Y. Wang , X. Wang , Cell Death Dis. 2019, 10, 171. 30787270 10.1038/s41419-019-1417-4 PMC6382841

[44] X. Wang , Z. Lu , A. Gomez , G. C. Hon , Y. Yue , D. Han , Y. Fu , M. Parisien , Q. Dai , G. Jia , B. Ren , T. Pan , C. He , Nature 2014, 505, 117. 24284625 10.1038/nature12730 PMC3877715

[45] A. Kechin , U. Boyarskikh , A. Kel , M. Filipenko , J. Comput. Biol. 2017, 24, 1138. 28715235 10.1089/cmb.2017.0096

[46] D. Kim , B. Langmead , S. L. Salzberg , Nat. Methods 2015, 12, 357. 25751142 10.1038/nmeth.3317 PMC4655817

[47] Y. Zhang , T. Liu , C. A. Meyer , J. Eeckhoute , D. S. Johnson , B. E. Bernstein , C. Nusbaum , R. M. Myers , M. Brown , W. Li , X. S. Liu , Genome Biol. 2008, 9, R137. 18798982 10.1186/gb-2008-9-9-r137 PMC2592715

[48] Z. Li , H. Weng , R. Su , X. Weng , Z. Zuo , C. Li , H. Huang , S. Nachtergaele , L. Dong , C. Hu , X. Qin , L. Tang , Y. Wang , G.‐M. Hong , H. Huang , X. Wang , P. Chen , S. Gurbuxani , S. Arnovitz , Y. Li , S. Li , J. Strong , M. B. Neilly , R. A. Larson , X. Jiang , P. Zhang , J. Jin , C. He , J. Chen , Cancer Cell 2017, 31, 127. 28017614 10.1016/j.ccell.2016.11.017 PMC5234852

[49] P.‐A. Yao , Y. Wu , K. Zhao , Y. Li , J. Cao , C. Xing , Cell Death Dis. 2022, 13, 103. 35110552 10.1038/s41419-022-04554-w PMC8810793

