# Supplementary material for: METTL3 Mediates Epithelial–Mesenchymal Transition by Modulating FOXO1 mRNA N6‐Methyladenosine‐Dependent YTHDF2 Binding: A Novel Mechanism of Radiation‐Induced Lung Injury
Source: Adv Sci (Weinh). 2023 Apr 18;10(17):2204784. doi: 10.1002/advs.202204784 (PMC10265050; doi:10.1002/advs.202204784)
Supplement: Supplementary file 1 — Supporting Information [file ADVS-10-2204784-s001.pdf]

ADVANCED SCIENCE
Open Access

Supporting Information

for *Adv. Sci.*, DOI 10.1002/advs.202204784

METTL3 Mediates Epithelial–Mesenchymal Transition by Modulating FOXO1 mRNA N^6-Methyladenosine-Dependent YTHDF2 Binding: A Novel Mechanism of Radiation-Induced Lung Injury

Yang Feng, Ping Yuan, Hongjuan Guo, Liming Gu, Zhao Yang, Jian Wang, Wei Zhu, Qi Zhang, Jianping Cao, Lili Wang and Yang Jiao**

Supporting Information for

METTL3 Mediates Epithelial–Mesenchymal Transition by Modulating FOXO1 mRNA N^6-Methyladenosine-Dependent YTHDF2 Binding: A Novel Mechanism of Radiation-Induced Lung Injury

Yang Feng, Ping Yuan, Hongjuan Guo, Liming Gu, Zhao Yang, Jian Wang, Wei Zhu, Qi Zhang, Jianping Cao, Lili Wang, Yang Jiao**

The file includes:

1. Supplementary Materials and Methods
2. Supplementary Figures and Figure Legends
3. Supplementary Tables

Supplementary Materials and Methods

Reagents and Materials

Dimethyl sulfoxide (DMSO) was purchased from Solarbio (Beijing, China). Evans blue dye was purchased from Sigma–Aldrich (St. Louis, MO, USA). LY294002 was obtained from MedChemExpress (Monmouth Junction, NJ, USA). SCH772984 was purchased from Selleck (Houston, TX, USA). Actinomycin D was obtained from GlpBio (Montclair, CA, USA). METTL3-targeting siRNA, FOXO1-targeting siRNA, YTHDF1-targeting siRNA, YTHDF2-targeting siRNA and YTHDF3-targeting siRNA were obtained from Santa Cruz Biotechnology (Santa Cruz, CA, USA). The control adenovirus (Ad-NC), adenovirus expressing METTL3 (Ad-METTL3) and adenovirus expressing ALKBH5 (Ad-ALKBH5) were purchased from Vigene Biosciences (Jinan, China). Antibodies against GAPDH and α-tubulin, and HRP-conjugated anti-mouse and anti-rabbit immunoglobulin G (IgG) were purchased from Beyotime (Nantong, China). Enzyme-linked immunosorbent assay (ELISA) kits for mouse IL-1β, IL-6, TGF-β and tumor necrosis factor-α (TNF-α) were purchased from BlueGene (Shanghai, China). The Dynabead mRNA Purification Kit (#61006) was purchased from Invitrogen (Carlsbad, CA, USA). An m^6A RNA Methylation Quantification Kit (#ab185912) and antibodies against Slug (#ab27568), ALKBH5 (#ab195377), Vimentin (#ab92547) and CREB3 (#ab180119) were purchased from Abcam (Cambridge, MA, USA). Antibodies against CD31 (#3528), AKT (#4691), phospho-AKT (p-AKT) (#4060), ERK1/2 (#4695) and phospho-ERK1/2 (p-ERK) (#4370) were purchased from Cell Signaling Technology (Beverly, MA, USA). Antibodies against VE-cadherin (#YT5611), E-cadherin (#YT1454), TGF-β1 (#YT4632), Snail1 (#YT4351)

and myeloperoxidase (MPO) (#YT5351) were purchased from Immunoway (Newark, DE, USA). Antibodies against METTL3 (#CY7240), α-SMA (#CY5295), PRKAA1 (#CY5326), PRKCA (#CY5363), AKT1S1 (#CY6904) and FOXO1 (#CY5376) were purchased from Abways (Shanghai, China). Antibodies against YTHDF1 (#17479-1-AP), YTHDF2 (#24744-1-AP), YTHDF3 (#25537-1-AP) and N-cadherin (#66219-1-Ig) were purchased from Proteintech (Chicago, IL, USA). Antibody against F4/80 (#GTX101895) was obtained from GeneTex (San Antonio, Texas, USA). Antibody against SEMA6B (#PA5-47251) was obtained from Invitrogen (Carlsbad, CA, USA). The human FOXO1-coding region (GenBank accession no. NM_002015.4) and human YTHDF2-coding region (GenBank accession no. NM_016258.3) were amplified by PCR using primer pairs specific for FOXO1 and YTHDF2, respectively. The amplified fragments were inserted into a pEnCMV vector. The plasmids were then sequenced by PPL Biotech (Nanjing, China) for confirmation.

Western Blot Analysis

The sources of the antibodies used for Western blot analysis are provided in the Reagents and Materials section. Western blot analysis was used to detect the expression of E-cadherin, VE-cadherin, CD31, N-cadherin, Vimentin, α-SMA, Snail1, Slug, METTL3, ALKBH5, FOXO1, SEMA6B, PRKAA1, PRKCA, CREB3, AKT1S1, YTHDF1, YTHDF2, YTHDF3, AKT, phosphorylated AKT (p-AKT), ERK1/2, phosphorylated ERK1/2 (p-ERK1/2), GAPDH and α-Tubulin in pulmonary tissues and cell lines. In brief, cells and tissue were lysed in RIPA buffer containing protease inhibitor and phosphatase inhibitor. After centrifugation at 4°C for 15 min (12,000 x g), the supernatant was collected for

Western blot analysis. Proteins were fractionated by 10% SDS–PAGE, transferred to polyvinylidene difluoride membranes (EMD Millipore, Bedford, MA, USA) and probed overnight at 4°C with the following primary antibodies: anti-E-cadherin (1:1000), anti-VE-cadherin (1:1000), anti-CD31 (1:1000), anti-N-cadherin (1:2000), anti-TGF-β1 (1:1000), anti-Vimentin (1:1000), anti-α-SMA (1:1000), anti-Snail1 (1:1000), anti-Slug (1:1000), anti-METTL3 (1:1000), anti-ALKBH5 (1:1000), anti-FOXO1 (1:1000), anti-SEMA6B (1:1000), anti-PRKAA1 (1:1000), anti-PRKCA (1:1000), anti-CREB3 (1:1000), anti-AKT1S1 (1:1000), anti-YTHDF1 (1:1000), anti-YTHDF2 (1:1000), anti-YTHDF3 (1:1000), anti-AKT (1:1000), anti-p-AKT (1:2000), anti-ERK1/2 (1:1000), anti-p-ERK1/2 (1:1000), anti-GAPDH (1:1000) and anti-α-Tubulin (1:1000). The membranes were washed with PBST and incubated with HRP-conjugated secondary antibodies (goat anti-mouse or goat anti-rabbit, 1:3000; Beyotime, Nantong, China) for 1 h at room temperature. After washing with PBST, the blots were incubated with enhanced chemiluminescence regent (ECL; Beyotime, Nantong, China) and then detected with a FluorChem™ M system (Protein Simple, San Jose, CA, USA).

Quantitative Real-Time Polymerase Chain Reaction (qRT–PCR)

Total RNA was extracted from cells or tissues using TRIzol reagent (Invitrogen, Carlsbad, CA, USA). RNA was reverse transcribed into cDNA using a PrimeScript RT kit (Takara, Shiga, Japan) according to the manufacturer’s instructions. qRT–PCR was performed using a SYBR Green Master Mix Kit (Takara, Shiga, Japan) on an ABI ViiA 7 Real-Time PCR system (Applied Biosystems, Foster City, CA, USA). The sequences of the specific primer pairs are listed in Supplementary Table 1. Relative mRNA expression levels were

calculated by the $2^{-\Delta\Delta}$CT method with normalization to GAPDH or β-actin.

Immunofluorescence Assay

Cells were fixed with 4% formaldehyde and blocked with 3% bovine serum albumin (BSA) for 1 h at room temperature. The cells were incubated with antibodies against E-cadherin (1:200), VE-cadherin (1:100), CD31 (1:3000), Vimentin (1:250) and α-SMA (1:100) overnight at 4°C and then with a Cy3-conjugated secondary antibody (goat anti-mouse or goat anti-rabbit, 1:1000; Beyotime, Nantong, China) for 1 h at room temperature. 4',6-diamidino-2-phenylindole (DAPI) was used for nuclear staining, and images were acquired under an FV1200 confocal microscope (Olympus, Tokyo, Japan).

Luciferase Reporter Assay

Cells were transfected with luciferase reporter and internal control pRL-TK (Promega, Madison, WI, USA) using ExFect transfection reagent (Vazyme, Nanjing, China). Luciferase activity was detected using a Dual-Luciferase Reporter Assay System. Promoter activities were expressed as the ratio of *Firefly* luciferase activity to *Renilla* luciferase activity as previously described.[1]

The following sequences were used:

FOXO1 with WT m^6A sites:

5'-TTCAGGCCCTGGTTGGGCAGGAAAGTGATGTATAGTTATGGACACTTTGCGTTT
CTTATTTAGGATAACTTAATATGTTTTTATGTATGTATTTTAAAGAAATTTCATCTGCTTCT
ACTGAACTATGCGTACTGCATAGCATCAAGTCTTCTCTAGAGACCTCTGTAGTCCTGG
GAGGCCTCATAATGTTTGTAGATCAGAAAAGGGAGATCTGCATCTAAAGCAATGGTCC
TTTGTCAAACGAGGGATTTTGATCCACTTCACCATTTTGAGTTGAGCTTTAGCAAAAG

TTTCCCCTCATAATTCTTTGCTCTTGTTTCAGTCCAGGTGGAGGTTGGTTTTGTAGTT
CTGCCTTGAGGAATTATGTCAACACTCATACT-3’

FOXO1 with mutated m^6A sites:

5’-TTCAGGCCCTGGTTGGGCAGGAAAGTGATGTATAGTTATGGCCACTTTGCGTTT
CTTATTTAGGATAACTTAATATGTTTTTATGTATGTATTTTAAAGAAATTTCATCTGCTTCT
ACTGAACTATGCGTACTGCATAGCATCAAGTCTTCTCTAGAGACCTCTGTAGTCCTGG
GAGGCCTCATAATGTTTGTAGATCAGAAAAGGGAGATCTGCATCTAAAGCAATGGTCC
TTTGTCAAACGAGGGATTTTGATCCACTTCACCATTTTGAGTTGAGCTTTAGCAAAAG
TTTCCCCTCATAATTCTTTGCTCTTGTTTCAGTCCAGGTGGAGGTTGGTTTTGTAGTT
CTGCCTTGAGGAATTATGTCAACACTCATACT-3

shRNA Adeno-associated Virus (AAV) Production and Transfection

Four different mouse shRNAs for MELLT3 silencing were designed by Vigene Biosciences (Jinan, China). Two hundred microliters (5.0×10^{12} vp/mL) of AAV9 containing four METTL3 shRNAs or scrambled shRNAs were injected via the tail vein. Two weeks later, the mice were exposed to radiation as described above. The shRNA sequences are shown in Supplementary Table 2.

Hematoxylin and Eosin (H&E) Staining

Lung tissues were fixed in 10% neutral-buffered formalin and embedded in paraffin. Three-micrometer paraffin sections were deparaffinized and heat-treated with citrate buffer (pH = 6.0) for seven min following an epitope retrieval protocol. Lung sections were stained with H&E.[1]

Masson’s Trichrome Staining

The 3 μm-thick slices sectioned from the lung blocks of the experimental groups were incubated with xylene (2 × 5 min) and a descending alcohol series (100%, 90%, 80%, 70% and 50%) for deparaffinization. Next, a Masson's Trichrome Stain Kit (Solarbio, Beijing, China) was applied to the sections as previously described).[1]

Pulmonary Coefficient Calculation

The body weight and lung wet weight were measured at 1, 4, 8 and 16 weeks after irradiation. The ratio of the lung wet weight (g) to the body weight (kg) was used as the pulmonary coefficient.

Vascular Permeability Assessment

To analyze vascular leakage, Evans blue dye was injected into the tail vein.[2] The mice were euthanized 3 hours later and transcranially perfused with saline to remove the blood. Evans blue dye was extracted from the pulmonary interstitium after incubation in formamide at 65°C for 24 h. The concentration of Evans blue dye was measured by absorbance at 620 nm and normalized to the lung tissue weight.

Immunohistochemistry (IHC)

Lung tissues were fixed in 10% neutral-buffered formalin and embedded in paraffin. Three-micrometer paraffin sections were deparaffinized and heat treated with citrate buffer (pH = 6.0) for 7 min following an epitope retrieval protocol. Three-micrometer paraffin sections were incubated with antibodies against E-cadherin (1:150), N-cadherin (1:200), TGF-β1 (1:200), Vimentin (1:200), α-SMA (1:300), Snail1 (1:150), Slug (1:150), F4/80 (1:150) and MPO (1:150) at 4°C overnight, followed by incubation with a biotinylated secondary antibody (ZSGB-Bio, Beijing, China). IHC staining was visualized

with substrate solution containing diaminobenzidine (DAB) and hydrogen peroxide. Counterstaining was performed with hematoxylin.

Enzyme-Linked Immunosorbent Assay (ELISA)

After the body weight of the mice was measured, 1 mL of blood was obtained via cardiac puncture. The serum was collected and stored at -80°C for later analysis by ELISA. The serum levels of IL-1β, IL-6, TGF-β and TNF-α were determined using ELISA kits according to the manufacturer's instructions. The optical absorbance of the samples was measured at 450 nm.

References

[1] J. Cao, W. Zhu, D. Yu, L. Pan, L. Zhong, Y. Xiao, Y. Gao, Y. Jiao, Q. Zhang, J. Ji, H. Yang, S. Zhang, J. Cao, *Radiat Res* **2019**, *192*, 410.

[2] X. Lei, N. He, L. Zhu, M. Zhou, K. Zhang, C. Wang, H. Huang, S. Chen, Y. Li, Q. Liu, Z. Han, Z. Guo, Z. Han, Z. Li, *Antioxid Redox Signal* **2021**, *35*, 849.

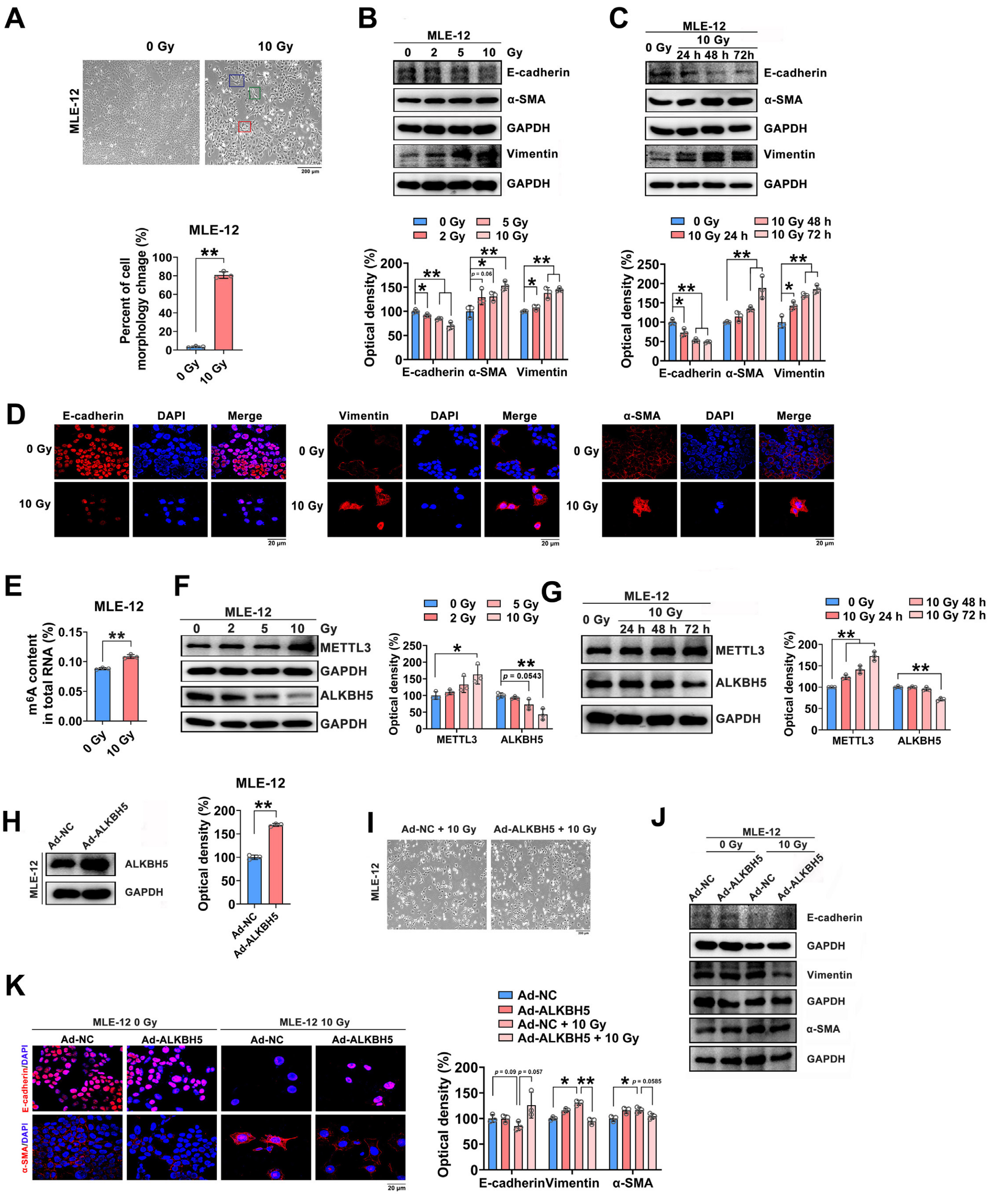

Supplementary Figure S1. The m⁶A RNA modification mediates IR-induced EMT during RILI in MLE-12 cells.

MLE-12 cells were exposed to 0, 2, 5 or 10 Gy X-rays. (A) Representative images of the cell morphology were obtained at 72 h after 0 or 10 Gy irradiation (scale bar = 200μm). In random microscope fields, the cells with morphologic changes were counted and the

percentage was calculated according to whether the cells became swollen (red outline), became elongated (green outline), or exhibited extended pseudopodia (blue outline) compared with a cuboidal appearance. The percentage was calculated (n = 3; mean ± SD; ***p* < 0.01; Student's *t-test*). (B) and (C) Western blot and quantitative analyses of EMT-related markers in MLE-12 cells (n = 3; mean ± SD; **p* < 0.05, ***p* < 0.01; Student's *t-test*). (D) Immunofluorescence staining of E-cadherin, α-SMA (red) and DAPI (blue) in MLE-12 cells at 72 h post-irradiation (scale bar = 20 μm). (E) The m^6A methylated RNA level was elevated in MLE-12 cells at 72 h after 10 Gy X-ray irradiation (n = 3; mean ± SD; ***p* < 0.01; Student's *t-test*). (F) Western blot and quantitative analyses of METTL3 and ALKBH5 protein levels at 72 h after different radiation doses (n = 3; mean ± SD; **p* < 0.05, ***p* < 0.01; Student's *t-test*). (G) The protein expression of m^6A modification enzymes in irradiated MLE-12 cells at different times (n = 3; mean ± SD; ***p* < 0.01; Student's *t-test*). MLE-12 cells were preinfected with control adenovirus or ALKBH5 adenovirus, followed by irradiation . (H) Western blot and quantitative analyses of ALKBH5 expression in MLE-12 cells (n = 3; mean ± SD; ***p* < 0.01; Student's *t-test*). (I) Phase contrast microscopy images of MLE-12 cells at 72 h after 10 Gy irradiation (scale bar = 200 μm). (J) Protein expression of EMT-associated markers at 72 h after 10 Gy irradiation (n = 3; mean ± SD; **p* < 0.05, ***p* < 0.01; Student's *t-test*). (K) Immunofluorescence was performed to detect the expression of E-cadherin and α-SMA in control or ALKBH5-overexpressing MLE-12 cells (scale bar = 20 μm).

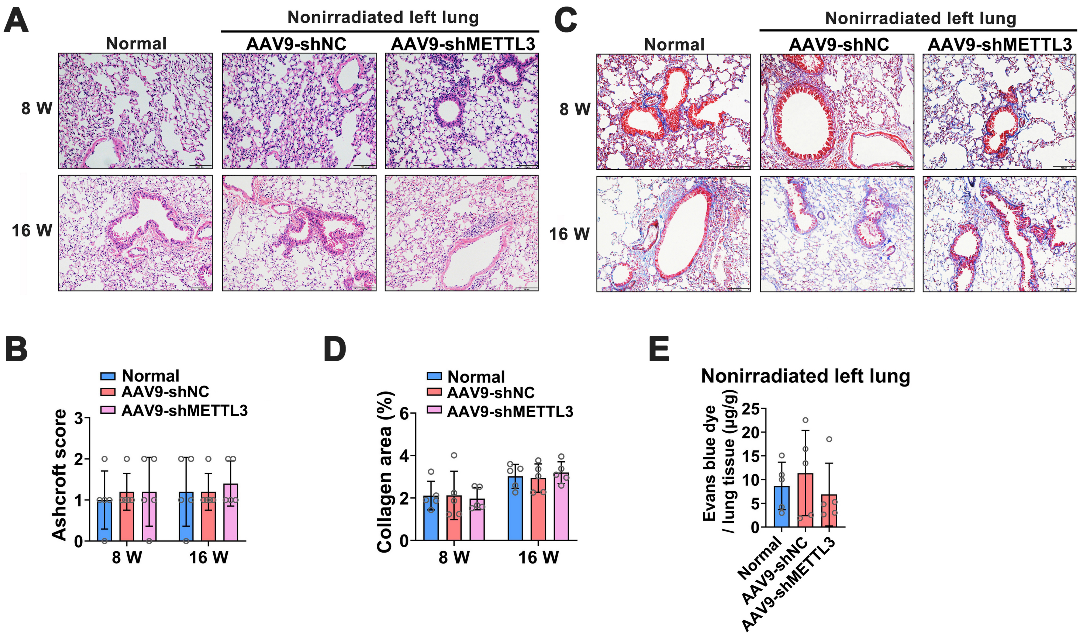

Supplementary Figure S2. METTL3 has no effect on the nonirradiated left lung.

The mouse right lung was not irradiated or irradiated with a single dose of 20 Gy X-rays, and the mice received AAV9-shNC or AAV9-shMETTL3 by tail vein injection two weeks before 20 Gy radiation. At 1, 8 and 16 weeks after irradiation, lung tissues were collected from the control and irradiated mice. (A) Representative H&E staining of mouse left lung tissues from various groups at 8 and 16 weeks after irradiation (scale bar = 100 μm). (B) A bar graph of the Ashcroft scores from H&E-stained slides of mouse left lung tissues (n = 5 mice per group; mean ± SD; ns: not significant; Student's *t-test*). (C) Representative Masson's trichrome staining of the mouse left lung (scale bar = 100 μm). (D) Quantification of the collagen deposition area in mouse left lung tissues (n = 5 mice per group; mean ± SD; ns: not significant; Student's *t-test*). (E) Evans blue staining was used to observe pulmonary microvascular injury (n = 5 mice per group; mean ± SD; ns: not significant; Student's *t-test*).

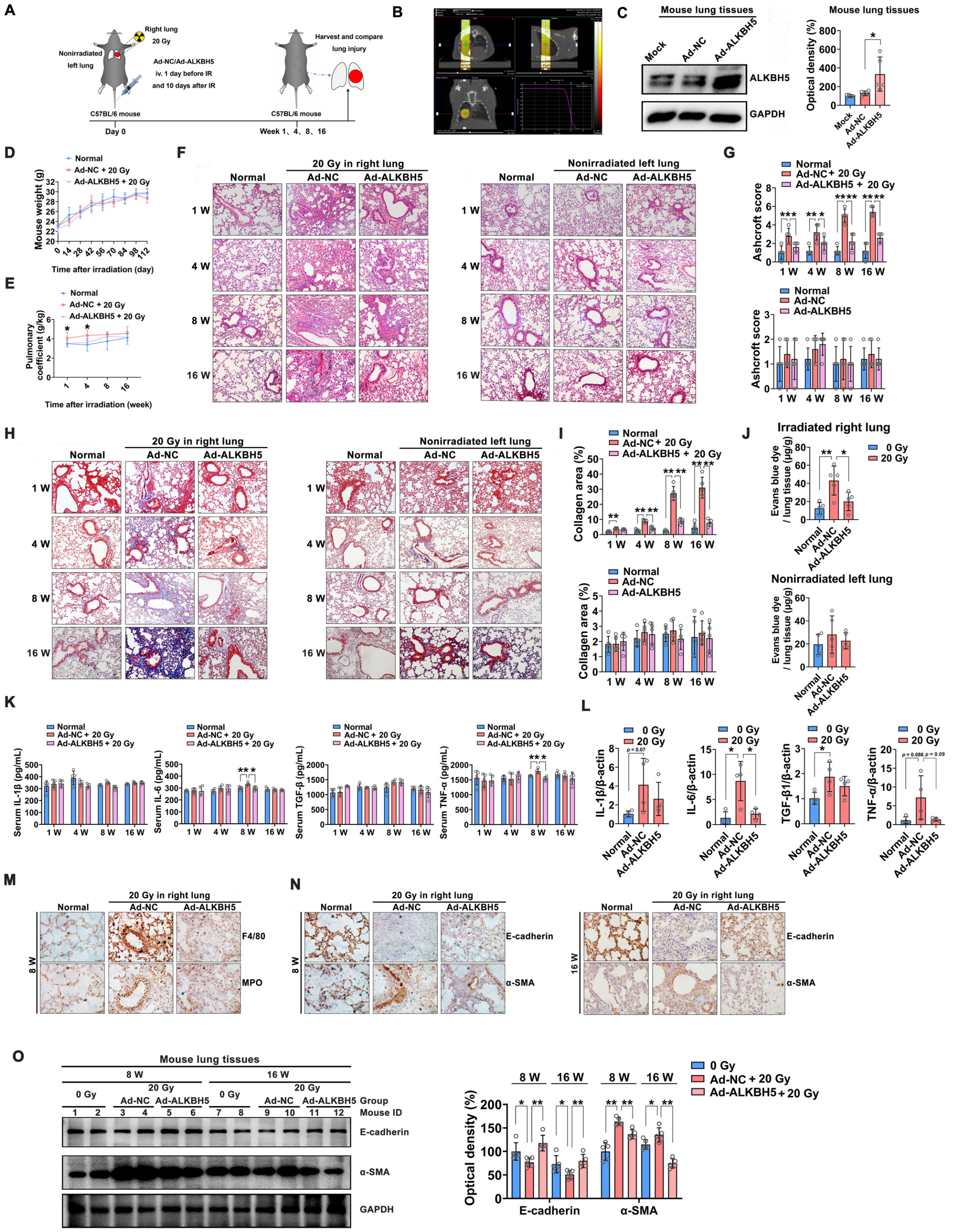

Supplementary Figure S3. The effect of ALKBH5 on RILI in mice.

(A) Experimental scheme of the mouse treatment protocol. The mouse right lung remained without irradiation or was irradiated with a single dose of 20 Gy X-ray irradiation, and the mice received Ad-NC or Ad-ALKBH5 by tail vein injection one day before 20 Gy

irradiation and ten days after 20 Gy irradiation. At 1, 4, 8 and 16 weeks after irradiation, lung tissues were collected from the control and irradiated mice (n =5 mice per group). (B) Target volume determination of the mice. (C) Western blot and quantitative analyses of ALKBH5 expression in the three groups of mouse lung tissues (n = 5 mice per group; mean ± SD; $*p < 0.05$; Student's *t-test*). (D) The mouse weight in each group was monitored from week 0 to week 16 (n = 5 mice per group; mean ± SD; ns: not significant; two-way ANOVA). (E) A line graph of the pulmonary coefficients from different groups of mice following local irradiation. Pulmonary coefficient = lung weight (g)/rat weight (kg) (n = 5 mice per group; mean ± SD; $*p < 0.05$; Student's *t-test*). (F) Representative H&E staining of mouse lung tissues from various groups at 1, 4, 8 and 16 weeks after irradiation (scale bar = 100 μm). (G) A bar graph of the Ashcroft scores from of H&E-stained slides of mouse lung tissues (n = 5 mice per group; mean ± SD; $*p < 0.05$, $**p < 0.01$; Student's *t-test*). (H) Representative Masson's trichrome staining of the lungs of the mice (scale bar = 100 μm). (I) Quantification of the collagen deposition area in the mouse lung tissues (n = 5 mice per group; mean ± SD; $**p < 0.01$; Student's *t-test*). (J) Evans blue staining was used to observe pulmonary microvascular injury (n = 5 mice per group; mean ± SD; $*p < 0.05$, $**p < 0.01$; Student's *t-test*). (K) ELISA of the serum levels of IL-6, IL-1β, TGF-β and TNF-α in different groups of radiation-induced injury mouse models at 1, 4, 8 and 16 weeks after irradiation (n = 5 mice per group; mean ± SD; $*p < 0.05$, $**p < 0.01$; Student's *t-test*). (L) qRT–PCR analysis of the IL-6, IL-1β, TGF-β and TNF-α mRNA levels in the right lung tissues of mice from different groups 8 weeks after irradiation (n = 4 mice per group; mean ± SD; $*p < 0.05$; Student's *t-test*). (M) Right lung tissues were immunostained for F4/80

and MPO and counterstained with hematoxylin (scale bar = 50 μm). (N) Immunohistochemistry staining of E-cadherin and α-SMA in right lung tissues from the three groups of mice 8 and 16 weeks after irradiation (scale bar = 50 μm). (O) Lung protein levels of the epithelial marker E-cadherin and interstitial markers Vimentin and α-SMA (n = 5 mice per group; mean ± SD; *$p < 0.05$, **$p < 0.01$; Student's *t-test*).

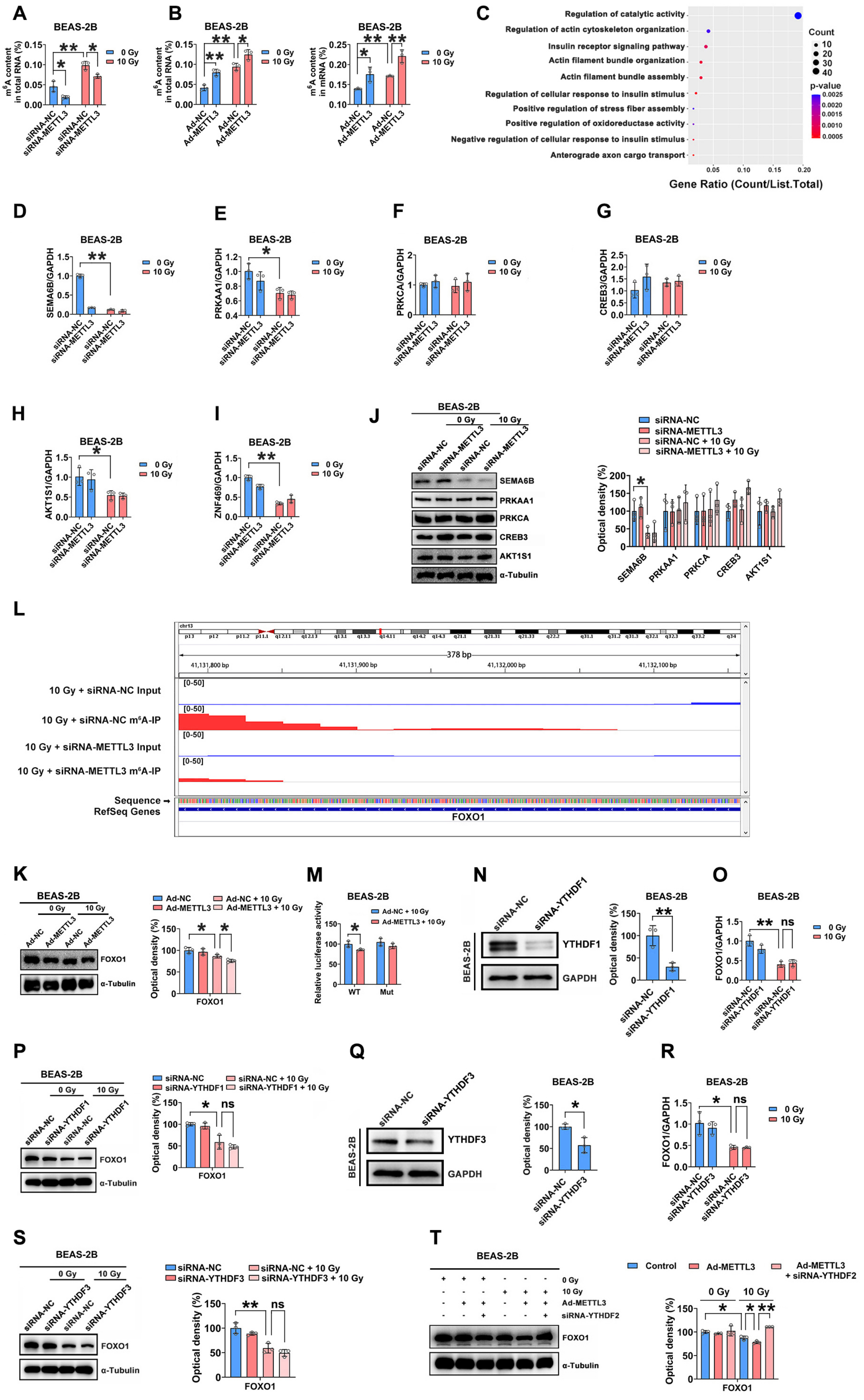

Supplementary Figure S4. METTL3-enhanced m^{6}A modification of FOXO1 mRNA and subsequent binding of YTHDF2 inhibit FOXO1 expression.

(A) The m^{6}A level in total RNA from BEAS-2B cells with or without METTL3 knockdown (n = 3; mean ± SD; *p < 0.05, **p < 0.01; Student's *t-test*). (B) The m^{6}A levels in total RNA and mRNA from BEAS-2B cells with or without METTL3 overexpression (n = 3; mean ± SD; *p < 0.05, **p < 0.01; Student's *t-test*). (C) GO analysis of hypomethylated m^{6}A transcripts (p < 0.05 was used as the threshold of significant enrichment). (D-I) qRT–PCR was performed in BEAS-2B cells with or without METTL3 silencing, followed by irradiation to validate the candidate genes (n = 3; mean ± SD; *p < 0.05, **p < 0.01; Student's *t-test*). (J) The protein levels of SEMA6B, PRKAA1, PRKCA, CREB3 and AKT1S1 in BEAS-2B cells (n = 3; mean ± SD; * < 0.05; Student's *t-test*). (K) The protein level of FOXO1 in BEAS-2B cells with or without METTL3 overexpression (n = 3; mean ± SD; *p < 0.05; Student's *t-test.* (L) m^{6}A abundance in the FOXO1 mRNA transcript (chr13:41131781-41132160). (M) Luciferase vectors with WT or mutated FOXO1 were transfected into BEAS-2B cells with or without METTL3 overexpression, followed by exposure to 0 or 10 Gy X-ray irradiation. Relative luciferase activity was detected (n = 3; mean ± SD; *p < 0.05; Student's *t-test*). (N) The YTHDF1 protein level in control and YTHDF1 knockdown BEAS-2B cells (n = 3; mean ± SD; **p < 0.01; Student's *t-test)*. BEAS-2B cells were transfected with or without siRNA-YTHDF1 prior to exposure to 0 or 0 Gy X-irradiation. The (O) mRNA and (P) protein levels of FOXO1 were measured (n =3; mean ± SD; *p < 0.05, **p < 0.01, ns: not significant; Student's *t-test*). (Q) The YTHDF3 protein level in control and YTHDF3 knockdown

BEAS-2B cells (n = 3; mean ± SD; *$p < 0.05$; Student's *t-test*). BEAS-2B cells were transfected with or without siRNA-YTHDF3 prior to exposure to 0 or 10 Gy X-irradiation. The (R) mRNA and (S) protein levels of FOXO1 were measured (n = 3; mean ± SD; *$p < 0.05$, **$p < 0.01$, ns: not significant; Student's *t-test*). (T) FOXO1 protein level in BEAS-2B cells treated with Ad-METTL3 and/or siRNA-YTHDF2 (n = 3; mean ± SD; *$p < 0.05$, **$p < 0.01$; Student's *t-test*).

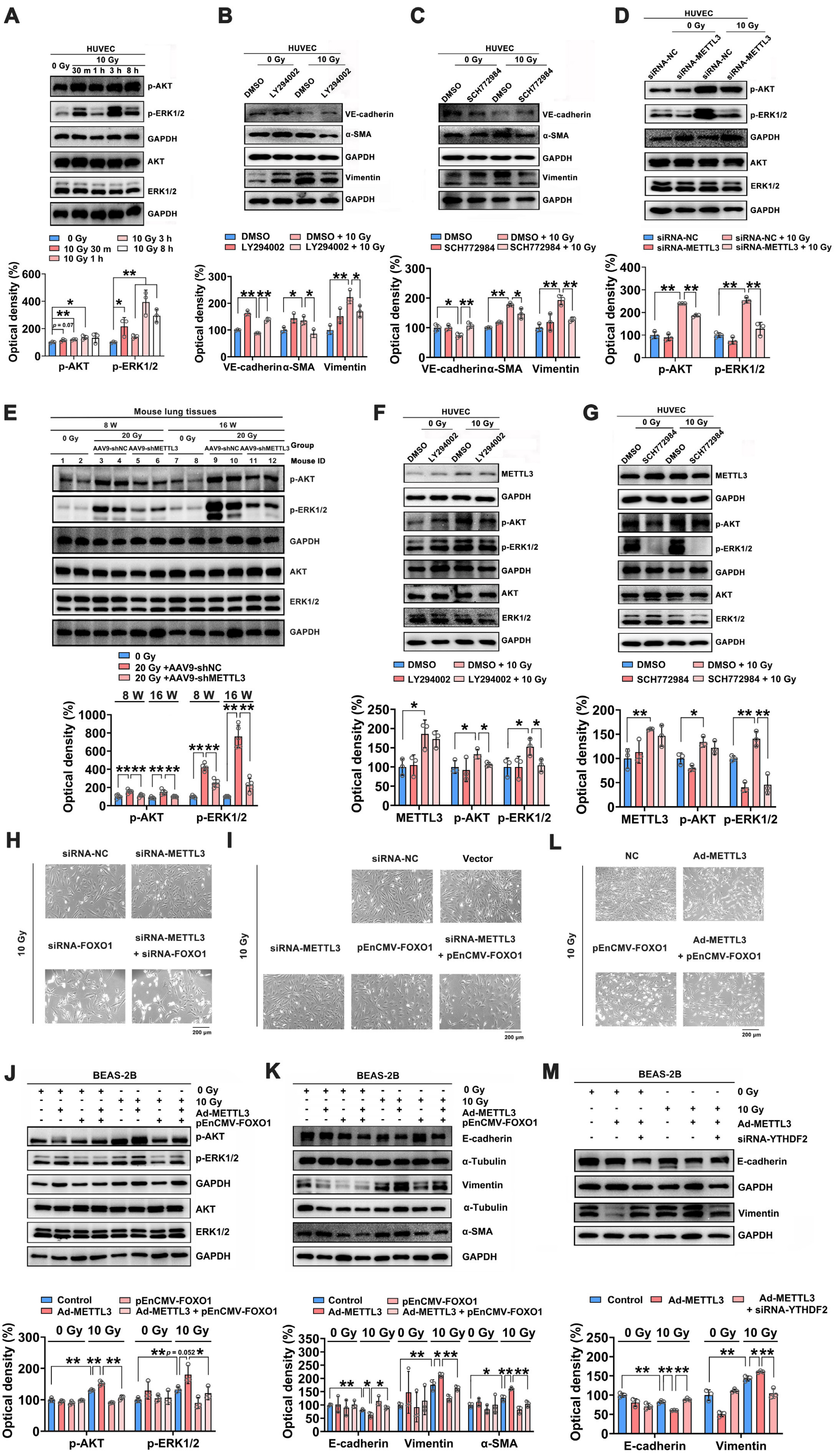
A
B
C
D
HUVEC
0 Gy
10 Gy
30 m
1 h
3 h
8 h
p-AKT
p-ERK1/2
GAPDH
AKT
ERK1/2
DMSO
LY294002
SCH772984
siRNA-NC
siRNA-METTL3
VE-cadherin
α-SMA
Vimentin
10 Gy 30 m
10 Gy 1 h
10 Gy 3 h
10 Gy 8 h
DMSO + 10 Gy
LY294002 + 10 Gy
SCH772984 + 10 Gy
siRNA-NC + 10 Gy
siRNA-METTL3 + 10 Gy
Optical density (%)
p = 0.07
E
Mouse lung tissues
8 W
16 W
20 Gy
AAV9-shNC
AAV9-shMETTL3
Group
Mouse ID
20 Gy +AAV9-shNC
20 Gy +AAV9-shMETTL3
F
G
METTL3
H
I
L
siRNA-FOXO1
siRNA-METTL3 + siRNA-FOXO1
Vector
pEnCMV-FOXO1
siRNA-METTL3 + pEnCMV-FOXO1
NC
Ad-METTL3
Ad-METTL3 + pEnCMV-FOXO1
200 μm
J
K
M
BEAS-2B
siRNA-YTHDF2
E-cadherin
α-Tubulin
Control
Ad-METTL3 + siRNA-YTHDF2
p = 0.052

Supplementary Figure S5. METTL3/FOXO1-dependent AKT-ERK activation is critical for IR-induced EMT.

(A) Western blot and quantitative analyses of AKT, phosphorylated AKT, ERK and phosphorylated ERK levels at different times after 0 or 10 Gy irradiation in HUVECs (n = 3; mean ± SD; *p < 0.05, **p < 0.01; Student's *t-test*). (B) HUVECs were incubated with or without the selective AKT inhibitor LY294002 (0.5 μM) for 2 h before 10 Gy irradiation. Western blot and quantitative analyses of VE-cadherin, Vimentin and α-SMA expression at 72 h post-irradiation (n = 3; mean ± SD; *p < 0.05, **p < 0.01; Student's *t-test*). (C) HUVECs were incubated with or without SCH772984 (0.5 μM) for 2 h before irradiation. The protein of EndMT-related markers were measured at 72 h post-irradiation (n = 3; mean ± SD; *p < 0.05, **p < 0.01; Student's *t-test*). (D) The protein expression of AKT, ERK, p-AKT and p-ERK in METTL3 knockdown HUVECs with or without exposure to 10 Gy irradiation (n = 3; mean ± SD; **p < 0.01; Student's *t-test*). (E) The protein expression of AKT, ERK, p-AKT and p-ERK in the lung tissues of RILI mouse model (n = 5 mice per group; mean ± SD; **p < 0.01; Student's *t-test*). (F and G) HUVECs were incubated with/without an AKT or ERK inhibitor for 2 h followed by exposure to 10 Gy irradiation. The protein expression of METTL3, AKT, ERK, p-AKT and p-ERK (n = 3; mean ± SD; *p < 0.05, **p < 0.01; Student's *t-test*). (H) Phase contrast microscopy images of BEAS-2B cells transfected with siRNA-METTL3 and/or siRNA-FOXO1 at 72 h after 10 Gy irradiation (scale bar = 200 μm). (I) Phase contrast microscopy images of BEAS-2B cells transfected with siRNA-METTL3 and/or FOXO1 overexpressing vector with or without radiation 72 h after 10 Gy irradiation (scale bar = 200 μm). (J) The protein levels of AKT, ERK, p-AKT

and p-ERK were measured in BEAS-2B cells treated with METTL3 adenovirus and/or FOXO1 plasmid with or without radiation (n = 3; mean ± SD; *p < 0.05, **p < 0.01; Student's *t-test*). (K) EMT-related protein levels in BEAS-2B cells treated with METTL3 adenovirus and/or FOXO1 plasmid with or without radiation (n = 3; mean ± SD; *p < 0.05, **p < 0.01; Student's *t-test*). (L) Phase contrast microscopy images of BEAS-2B cells supplemented with METTL3 adenovirus and/or FOXO1 plasmid at 72 h after 10 Gy irradiation (scale bar = 200 μm). (M) Western blot and quantitative analyses of E-cadherin and Vimentin in BEAS-2B cells treated with Ad-METTL3 and/or siRNA-YTHDF2 (n =3; mean ± SD; *p < 0.05, **p < 0.01; Student's *t-test*).

Supplementary Tables

Supplementary Table 1 Primers used for quantitative real-time polymerase chain reaction (qRT–PCR) amplification

Gene names	Primer names	Primer sequences
GAPDH	Forward	5'-CAGGAGGCATTGCTGATGAT-3'
(Rat)	Reverse	5'-GAAGGCTGGGGCTCATTT-3'
METTL3	Forward	5'-CTGGCACCCGAAAGATTGAG-3'
(Rat)	Reverse	5'-CTGTGCTTAAACCGGGCAAC-3'
METTL14	Forward	5'-ACCGTGAAGCGAAGCACAGATG-3'
(Rat)	Reverse	5'-TTCTATGTTGCCAATCTCAGGTTCCTC-3'
WTAP	Forward	5'-GCCTGGAAGTTTACGCCTGATAGC-3'
(Rat)	Reverse	5'-TCTTGGTTCTCCTGGATAAGCATTCG-3'
ALKBH5	Forward	5'-GGAAGTACCAGGAGGACTCAGACC-3'
(Rat)	Reverse	5'-GGATGCCGCTCTTCACCTTGC-3'
FTO	Forward	5'-GCTGTGGAAGAAGATGGAGAGTGTG-3'
(Rat)	Reverse	5'-ATCAGGACGGCAGACAGAATTTCAC-3'
β-actin	Forward	5'-CATCCGTAAAGACCTCTATGCCAAC-3'
(Mouse)	Reverse	5'-ATGGAGCCACCGATCCACA-3'
METTL3	Forward	5'-CGCTGCCTCCGATGTTGATCTG-3'
(Mouse)	Reverse	5'-TCTCCTGACTGACCTTCTTGCTCTG-3'
ALKBH5	Forward	5'-TGTTCTTGGCTTTCCTCCTTGATGG-3'
(Mouse)	Reverse	5'-TGTCTCTACTGGCTACTCTGGTGTG-3'
IL-1β	Forward	5'-AAATCTCGCAGCAGCACATCAA-3'
(Mouse)	Reverse	5'-CCACGGGAAAGACACAGGTAGC-3'
IL-6	Forward	5'-AGTTGCCTTCTTGGGACTGA-3'
(Mouse)	Reverse	5'-CCTCCGACTTGTGAAGTGGT-3'
TGF-β1	Forward	5'-TTGCTTCAGCTCCACAGAGA-3'
(Mouse)	Reverse	5'-TGGTTGTAGAGGGCAAGGAC-3'
TNF-α	Forward	5'-GACAAGGCTGCCCCGACTACG-3'
(Mouse)	Reverse	5'-CTTGGGGCAGGGGCTCTTGAC-3'
GAPDH	Forward	5'-GTCTCCTCTGACTTCAACAGCG-3'
(Human)	Reverse	5'-ACCACCCTGTTGCTGTAGCCAA-3'
YTHDF2	Forward	5'-ACTTCTCAGCATGGGGAAATAA-3'
(Human)	Reverse	5'-TATTCATGCCAGGAGCCTTATT-3'
FOXO1	Forward	5'-AAACACCAGTTTGAATTCACCC-3'
(Human)	Reverse	5'-TCGACTTATTGTCCTGAAGTGT-3'
SEMA6B	Forward	5'-CGGAGACAACATCAGCGGTATGG-3'
(Human)	Reverse	5'-AAGAGCATCCCGTCAGAGAAGAGG-3'
PRKAA1	Forward	5'-CAACTATCGATCTTGCCAAAGG-3'
(Human)	Reverse	5'-AACAGGAGAAGAGTCAAGTGAG-3'
PRKCA	Forward	5'-GGTGAAGGACCACAAATTCATC-3'
(Human)	Reverse	5'-CACCCGGACAAGAAAAAGTAAC-3'

CREB3	Forward	5'-TGGGAAGTAGATGATTTGCTGT-3'
(Human)	Reverse	5'-GTACTAGCCTAGCAATCTCCTG-3'
AKT1S1	Forward	5'-GTCATCAGATGAGGAGAATGGG-3'
(Human)	Reverse	5'-CTGGAAGTCGCTGGTGTTAAG-3'
ZNF469	Forward	5'-TCAGTCAATGCCAGTCCCAAAA-3'
(Human)	Reverse	5'-CTGCTTCCCTTGCATTTCTCTA-3'

Supplementary Table 2 Murine METTL3-targeting shRNA sequences

shRNA	Target sequences (mouse)
shMETTL3#1	5'-CCTCAGTGGATCTGTTGTGATTTCAAG AGAATCACAACAGATCCACTGAGGTTTTTT-3'
shMETTL3#2	5'-GCGTCAGTATCTTGGGCAAATTTCAAG AGAATTTGCCCAAGATACTGACGTTTTTT-3'
shMETTL3#3	5'-GCACCCGCAAGATTGAGTTATTTCAAG AGAATAACTCAATCTTGCGGGTGCTTTTTT-3'
shMETTL3#4	5'-GGAGATCCTAGAGCTATTAAATTCAAGA GATTTAATAGCTCTAGGATCTCCTTTTTT-3'
